# The effect of vagus nerve stimulation on heart rate and respiration rate and their impact on seizure susceptibility in anaesthetized rats under pentylenetetrazol

**DOI:** 10.3389/fnins.2025.1487082

**Published:** 2025-03-21

**Authors:** Javier Chávez Cerda, Elena Acedo Reina, Ayse S. Dereli, Louis Vande Perre, Romain Raffoul, Maxime Verstraeten, Michel-Antony Ngan Yamb, Enrique Germany Morrison, Elise Collard, Auriane Apaire, Pascal Doguet, Jérôme Garnier, Jean Delbeke, Riëm El Tahry, Antoine Nonclercq

**Affiliations:** ^1^Bio-, Electro-and Mechanical-Systems (BEAMS), Université Libre de Brussels, Brussels, Belgium; ^2^Institute of Neurosciences (IoNS), Université Catholique de Louvain, Brussels, Belgium; ^3^Synergia Medical SA, Mont-Saint-Guibert, Belgium

**Keywords:** vagus nerve stimulation, respiration rate, heart rate, autonomic system, seizure susceptibility

## Abstract

Despite the proven efficacy of vagus nerve stimulation (VNS) in seizure control, its precise mechanism of action remains unclear. VNS is known to impact the cardiorespiratory system. In this study, we explored the effects of standard and breathing-synchronized VNS on heart and respiratory rates in anesthetized epileptic rats, as well as their impact on seizure susceptibility. Seizures were induced in rats by intravenous pentylenetetrazol (PTZ) infusion. Three animal groups (*n* = 4) were subjected to different types of stimulation: Sham VNS, Standard VNS, and Breathing-Synchronized VNS. Measurements included respiration, electrocardiogram, electroencephalogram, and vagal electroneurogram. Each experiment began with a 5-min baseline period, followed by PTZ infusion until tonic–clonic seizure onset, confirmed by video recording and electroencephalogram. Results indicate that the stimulation significantly decreased the heart rate below baseline levels for standard VNS (−120.0 ± 69.1 bpm) and breathing-synchronized VNS (−84.9 ± 61.0 bpm), overcoming the heart rate increasing effect of PTZ infusion observed in the sham VNS (+79.2 ± 35.5 bpm), and there was no recovery during OFF periods. Regarding the breathing rate changes, the sham VNS group presented a slight increase with respect to baseline (+13.6 ± 1.8 bpm). The stimulation slightly increased the average breathing rate for standard VNS (+13.0 ± 14.6 bpm) and breathing-synchronized VNS (+13.7 ± 10.4 bpm), however with significantly enlarged standard deviation. More specifically, the breathing rate presented a pattern that suggests that the rats experienced respiratory hypoxia under stimulation. The VNS modulation of the heart rate and breathing rate in the standard VNS group was similar in the breathing-synchronized VNS, suggesting that the VNS effect is cumulative. Unexpectedly, the sham VNS group required a higher PTZ dose (79.7 ± 13.4 mg/kg) to reach tonic–clonic seizures compared to the standard VNS group (57.9 ± 9.8 mg/kg), and the breathing-synchronized VNS group (60.0 ± 8.7 mg/kg), pointing to an increased seizure susceptibility of VNS in this particular model. Additionally, the latency of the seizures was longer in the sham VNS (291.5 ± 84.4 s) compared to standard VNS (200.5 ± 59.5 s) and breathing-synchronized VNS (206.9 ± 66.0 s), meaning that the seizures under stimulation were starting earlier. A significant linear relationship was found between heart rate and respiratory rate changes, and seizure susceptibility (*R*^2^ = 0.62, *p*-value = 0.012). We hypothesize that the significant drop in heart rate and the presence of altered respiration patterns, such as apneas or changes in breathing rates, caused by VNS, are related to hypoxia and hypotension conditions, which could increase susceptibility to PTZ. Future investigations with larger sample sizes, incorporating blood pressure and oxygen saturation monitoring, are needed to sort out the role of hypoxia and hypotension as potential covariates affecting the seizure susceptibility caused by overstimulation. Such a finding would support the idea that VNS safety and efficacy require precise adjustments.

## 1 Introduction

Epilepsy is a long-term condition affecting the brain, characterized by a continuous inclination to have seizures (Beghi, 2020). It is one of the most common neurological diseases worldwide, affecting around 70 million people (Brodie et al., 2012). Epilepsy impacts the quality of life of patients, causing stigma and discrimination (Baker et al., 1997), and in rare cases, can lead to premature death (sudden unexpected death in epilepsy, SUDEP) (Beghi, 2020). Approximately 30 to 40% of patients do not respond positively to antiepileptic medication and are categorized as refractory cases (Raedt et al., 2018). While brain surgery might provide seizure relief, not all individuals meet the criteria to benefit from epilepsy surgery (Wiebe and Jette, 2012). Moreover, some patients experience only modest improvements after surgery (Englot et al., 2013). In cases where seizures remain inadequately controlled, alternative interventions based on neuromodulation can be considered.

Vagus nerve stimulation (VNS) is an alternative for epileptic patients who do not respond to medication and do not qualify for brain surgery. VNS consists of an implant that sends electrical pulses - monophasic, biphasic, or triphasic - to the cervical vagus nerve through fitted electrodes (Wheless et al., 2018). It reduces by at least 50% the seizure frequency after 2 years of implantation (Iimura et al., 2023) and is less invasive than brain stimulation. VNS parameters such as stimulus amplitude, pulse width, frequency, and duty cycle must be carefully tuned to optimize the therapy for each patient (Englot et al., 2016) while minimizing the side effects (e.g., hoarseness, dyspnea). The parameters used for stimulation vary between patients, with a typical stimulation frequency of 20–30 Hz, stimulation current of 1.0–3.0 mA, pulse width of 130–500 μs, and duty cycle of 10–30% (30–90 s ON, followed by 5 min OFF) (Johnson and Wilson, 2018; Panebianco et al., 2016). At a population level, modal target output current and duty cycle were reported to be 1.61 mA and 17.1%, respectively (Fahoum et al., 2022).

Despite its positive outcomes, the underlying mechanism of VNS remains poorly understood (Groves and Brown, 2005). Besides its therapeutic effects, some side effects may result from unintended activation of off-target structures or undesired impact on autonomic functions. For example, VNS activates fibers of the vagus nerve, which innervate the heart (Olshansky et al., 2008) and the central respiratory populations (Molkov et al., 2014) within the brainstem. Consequently, VNS can present cardiac or respiratory complications. Although these complications rarely become significant in humans, there are instances of asystole (in 0.1% of patients), mainly during surgery (Ardesch et al., 2007), and respiratory issues predominantly during sleep (Parhizgar et al., 2011). Thus, it is crucial to use animal models to understand the mechanism of action of VNS and reduce the undesired effects, as clinical benefits observed in animal studies are often translated into human clinical trials. Studies of VNS in rat models have shown a pronounced impact on the cardiorespiratory system (Woodbury and Woodbury, 1990; McLachlan, 1993; Ahmed et al., 2020; Zaaimi et al., 2008; McAllen et al., 2018; Yaghouby et al., 2020). Woodbury and Woodbury (1990) and McLachlan (1993) reported that respiration stopped, heart rate dropped dramatically during the stimulation, and both resumed erratically when stimulation ceased. Hotta et al. (2009) also reported bradyarrhythmia and cardiac arrest in healthy rats. Additionally, Zaaimi et al. (2008) discussed the effects of different stimulation parameter combinations on heart rate, respiratory rate, and inspiratory strength in awake healthy rats, reporting different drops in heart rate and respiratory rate for different stimulation parameters.

Research on VNS has suggested that synchronizing stimulation with physiological variables (De Faria et al., 2024; Plachta et al., 2016) could alter the impacts on autonomic functions. The exhalation phase of breathing may be an appropriate timing to reduce the impact of VNS. Timing VNS to coincide with the exhalation phase takes advantage of the body’s natural parasympathetic activity during this period, which is associated with relaxation and reduced cardiovascular stress. By stimulating during exhalation, we hypothesized that VNS could be more seamlessly integrated into the body’s physiological processes, potentially reducing impacts on autonomic functions such as abrupt heart rate and respiratory rate changes that can occur when stimulation is misaligned with breathing patterns.

In this study, we aimed to explore the impact of two different VNS implementations on the autonomic system, specifically heart and respiratory rates in anesthetized rats: standard VNS and breathing-synchronized VNS. Secondly, we aimed to study the consequent effect of these autonomic changes on seizure susceptibility, by infusing intravenously pentylenetetrazol (PTZ) during the stimulation and calculating the PTZ dose needed to reach the tonic–clonic stage [PTZ threshold (Mandhane et al., 2007)].

## 2 Materials and methods

### 2.1 Data acquisition system

For this experiment, the respiration, the electrocardiogram (ECG), the electroencephalogram (EEG), and the vagus nerve electroneurogram (VENG) were measured. The signals were digitized with a NI-DAQ USB-6212 multifunction I/O-device (National Instruments, Austin, USA) for an overall resolution of 0.176 μV/bit for ECG and VENG, and 17.6 μV/bit for the respiration with a dynamic range of ±5.77 mV for ECG, EEG and VENG, and ± 115.4 mV for respiration (referred to the input), using a sampling frequency of 50 kS/s per channel. The acquisition system was previously validated (Stumpp et al., 2020; Chávez Cerda et al., 2022; Smets et al., 2021).

For visual monitoring of the experiment and the seizure development characterization (baseline, pre-ictal, seizure onset, and ictal-tonic–clonic), a smartphone video camera was used (Samsung Galaxy A02s, 30 fps, 1920 × 1,080 resolution). To synchronize the video recording and the signal acquisition, an onset and end marking trigger pulse was recorded, which also turned on a light-emitting diode placed in the field of the camera.

### 2.2 Stimulation

The stimulator consists of a controlled current source, used previously for in-vivo and ex-vivo studies (Chávez Cerda et al., 2022; Cury et al., 2021) to provide a biphasic, charge-balanced, with a cathodic-leading phase stimulation. Each phase (cathodic first, followed by anodic) had a pulse width of 500 μs and an amplitude of 500 μA. There was an interphase delay of 50 μs. High stimulation amplitude and large pulse width were chosen to address the VNS impact on the autonomic system. These parameters are, therefore, higher than the electrical dosage to address the antiepileptic effect that involves the activation of A and B fibers (Woodbury and Woodbury, 1990; McAllen et al., 2018; Krahl et al., 2001). The duty cycle was set to 50% (30 s ON, 30 s OFF). With those parameters, two stimulations were tested in this experiment: the standard VNS and the breathing-synchronized VNS. The standard VNS has a frequency of 20 Hz, and it delivers the pulses during all ON stimulation periods. The breathing-synchronized VNS is delivered at 40 Hz during each exhalation only during the ON time. The number of pulses given during VNS (linked to the total charge displaced or energy delivered) significantly impacts the autonomic functions (Fisher et al., 2021). To avoid any bias, we chose to have an equal number of pulses in both VNS paradigms (breathing-synchronized and standard VNS). Due to the stimulation exclusively occurring during exhalation in the breathing-synchronized VNS, the delivered pulses (or dose) are inherently fewer than in a standard stimulation continuously delivering pulse trains at 20 Hz. Its stimulation frequency was, therefore, increased to be comparable to the standard VNS. Assuming equal durations for inhalation and exhalation, a stimulation frequency of 40 Hz was chosen exclusively during exhalation, with its duration set at half the preceding inhalation-exhalation time.

A microcontroller (ESP32) measures and processes the respiration signal in real time for exhalation detection. An external Analog to Digital Converter (ADC) (Texas Instrument ADS8688IDBT, 16-bit resolution), connected to the microcontroller, was used to acquire the respiration signal at 1 kS/s. A real-time digital low pass filter (second order Butterworth IIR filter, cut-off frequency 10 Hz) was used for noise rejection. A 10-sample buffer was used to save the incoming data. An inspiration is detected when each buffer sample equals or is greater than the precedent sample. If not, an exhalation is detected, triggering stimulation. This stimulation setup for the breathing-synchronized VNS was already validated (Chávez Cerda et al., 2024), giving high accuracy on exhalation detection and a comparable stimulation dosage (pulses/s) to the standard VNS.

### 2.3 Seizure induction model

Rats were used as acute animal models, and seizures were induced using PTZ, which is a widely recognized chemical method to screen the activity of anticonvulsive treatments (Kandratavicius et al., 2014; Aalbers et al., 2011; Tseng et al., 2020; García-Cabrero et al., 2014). An intravenous (i.v.) PTZ infusion with a constant flow rate through the tail vein elicits seizure response in a reliable, reproducible, and rapid method. Screening of seizure development by the i.v. PTZ test provided insight into the seizure susceptibility and different phases of seizures in individual animals. Acute seizures were obtained by i.v. infusion (Aladdin Single-Syringe Pump-AL-300, World Precision Instrument, USA) of 10 mg/mL PTZ (MERK KGaA Darmstadt, Germany), diluted in physiological saline, in the lateral tail vein, at a rate of 0.5 mL/min/kg.

### 2.4 Surgical procedure

This experimental procedure was approved by the University Health Sciences Sector Laboratory Animal Protection Committee (2018/UCL/MD/001). An acute experimentation was performed on 12 male Wistar rats (3.1 ± 0.8 months, 355.4 ± 25.8 g, Refer to Supplementary Table 1). The rats were injected with Xylazine 7 mg/kg and Ketamine 100 mg/kg intraperitoneally. To maintain anesthesia, half of the initial dose was administered as soon as a withdrawal reflex was observed in response to a noxious mechanical stimulus applied to the paw. A tripolar microcuff electrode (Microprobe), 4 mm contact distance, was implanted in the cervical left vagus nerve to record the VENG signal. For the EEG signal acquisition, screw epidural electrodes were implanted on the scalp (AP 6 mm, ML 0 mm (reference), AP 2 mm, ML 3 mm (front left), AP 2 mm, ML −3 mm (front right), respect to bregma location). A reference ground for the EEG and VENG signal was placed on the scalp, using an additional screw epidural electrode (AP -5 mm, ML 3 mm). The ECG was acquired using three subcutaneous tungsten needle electrodes (ECG derivation II), placed on the paws. The respiration signal was acquired with a thermistor inside a silicone-made facemask.

Three groups of anesthetized rats (*n* = 4) were recorded: (i) Sham VNS, (ii) Standard VNS, and (iii) Breathing-synchronized VNS. A VENG, ECG, and respiration baseline recording were performed for 5 min. Then, the already implanted microcuff (on the vagus nerve) was connected to the stimulator. The central electrode was connected to the anode (source), and the rostral electrode was connected to the cathode (sink). To ensure proper electrode-nerve contact for the stimulation, the impedance between the central and rostral electrode contact was measured (IMP-2A - Single channel, Microprobes for Life Science, USA), giving an average impedance of 15.5 ± 4 kΩ. Right after, the stimulation was turned on, except for the rats under sham VNS. At the same time, the i.v. PTZ infusion started with the stimulation. The PTZ infusion was stopped when the rat reached the tonic–clonic stage. Finally, the recovery period was recorded for at least 5 min, while the VNS remained active.

### 2.5 Data processing

All calculations and post-processing were performed in Matlab 2023b (Mathworks). The EEG, ECG, and the respiration were down sampled from 50 kS/s to 1 kS/s.

For the VENG signal validation, we assessed whether the VENG signal exhibited an amplitude modulation synchronous to respiration during baseline for all the rats, confirming the functional integrity of the vagus nerve. The VENG signal was filtered using a band-pass 2^nd^ order Butterworth IIR filter 300–3,000 Hz, with zero-shift phase. The absolute median envelope of the filtered VENG signal was computed using a moving window of 12,500 samples (0.25 s), in steps of 1 sample (20 μs), resulting in 12,499 samples overlap, to extract the VENG respiratory burst. No boundary handling was used. Identical main frequency and constant phase difference are required if signals are fully synchronous. We performed a fast-Fourier transform (FFT) on both the respiration signal and the VENG envelope, comparing their dominant frequency peaks. Additionally, the mean Phase Locking Index (PLI) between the respiration signal and the VENG envelope was computed, following drift removal using a second-order Butterworth high-pass filter (0.1 Hz). The PLI quantifies how consistently the phase difference between two signals remains stable over time, where 0 indicates no phase synchronization and 1 represents a perfectly constant phase difference.

To assess changes in anesthesia level, the proportion of EEG delta-band energy was computed, relative to the total energy. Deeper anesthesia is associated with increased energy in the EEG’s delta frequency band (0.5–4 Hz) (Li et al., 2024). The EEG signals were filtered using a 2nd order Butterworth bandpass filter (0.5–100 Hz) with zero phase shift. The EEG signals were segmented into 5-s windows with a 1-s step, resulting in a 4-s overlap between consecutive windows. The FFT was computed for each window, using a rectangular window. The energy was calculated by summing the squared magnitudes of the FFT bins, with delta-band energy obtained by summing the bins corresponding to 0.5–4 Hz, while total energy was derived from all bins in the 0.5–100 Hz range. The proportion of delta-band energy relative to total energy was then determined during the baseline and infusion periods before the seizure onset (pre-ictal). Since the stimulation was applied during the infusion, the delta wave energy was discarded during ON periods to avoid stimulation artifact contamination. The EEG delta band energy proportion change between baseline and pre-ictal was computed.

For heart rate and breathing rate computation, the Matlab function “findpeaks” was applied to the ECG and respiration signals, respectively. For heart rate calculation, a minimum peak height was visually determined for each rat to account for stimulation artifacts, and a minimum peak-to-peak distance of 0.1 s was set to filter physiologically implausible detections. For respiratory rate, a minimum peak prominence threshold was set visually per rat to ensure reliable detection of true respiratory cycles. The detected peaks were then visually inspected and manually corrected for false positives and false negatives, and the instantaneous heart rate and breathing rate were computed. Then, they were then filtered using a moving mean filter (5-s window size, 1-s step, resulting in a 4-s overlap, chosen to balance temporal resolution and noise reduction). To compute the heart rate and breathing rate changes, the means of heart and breathing rates during the infusion were compared to the mean of the last 30 s of the baseline recording. Additionally, the mean heart rate and respiratory rate were calculated for a 2-min period, starting 3 min after the infusion stopped to assess the physiological changes following seizure activity. Respiratory periods exceeding 3 s were identified and classified as apneas. The maximal duration of these apneas was assessed during each stimulation period and tracked over time for each rat to evaluate potential habituation effects.

The autocorrelation function (ACF) was computed to assess the periodicity of heart rate and breathing rate time series induced by VNS, with drift removed using a zero-shift 2^nd^ order Butterworth high-pass filter (0.05 Hz). Additionally, the root mean square of successive differences (RMSSD) of the instantaneous heart and the respiration periods were computed to quantify the heart rate and breathing rate variability caused by VNS.

For assessing seizure susceptibility, the PTZ dose to reach tonic–clonic was calculated for each rat to determine the PTZ threshold. Additionally, the seizure latency was calculated per rat by computing the time from the start of the infusion to the appearance of gamma sharp spikes (30 Hz – 100 Hz) on the EEG signal, which indicates the seizure onset. The camera recording was also used simultaneously to help with the seizure onset detection, by looking at when the rat exhibited the first stages of the seizure (the total whisker extension or the presence of myoclonic jerks). Also, the seizure duration was calculated per rat in two phases: the ictal duration, measured from seizure onset to the end of infusion (when tonic–clonic activity started), and the post-ictal duration, measured from the end of infusion until the end of EEG depression.

For assessing the link between physiological changes and seizure susceptibility, Pearson’s linear correlation coefficient was calculated between the PTZ threshold and the age and weight of the rats. In addition, a multiple linear regression (“regress”), using least squares, allowed to determine if there was a linear relationship between the PTZ threshold and the heart and breathing rate changes, the baseline heart rate and breathing rate and the variability of heart rate and breathing rate.

### 2.6 Statistic tools

The Shapiro–Wilk test was used to test the normality of the samples. For statistical parametric average comparison and variance comparison between groups, the Student’s *t*-test (comparing means and paired) and the equal variance *F*-test were used when the data followed a normal distribution. For comparison in the same group, the pair-*t* test comparison was applied. A Wilcoxon signed-rank test was used for non-parametric comparison. For all the statistical tests, a significance level of *α* = 0.05 was used, and for multiple comparisons, Bonferroni correction was applied. Additionally, for practical significance, the size effect was computed (Cohen’s *d*) for multiple comparisons. Linear regression was used to identify potential relationships between physiological changes and seizure susceptibility. The interquartile range (IQR) method was applied to evaluate deviations or unusual trends in the dataset. All these statistical tools were provided by Matlab.

## 3 Results

### 3.1 VENG signal validation

**Figure 1:**
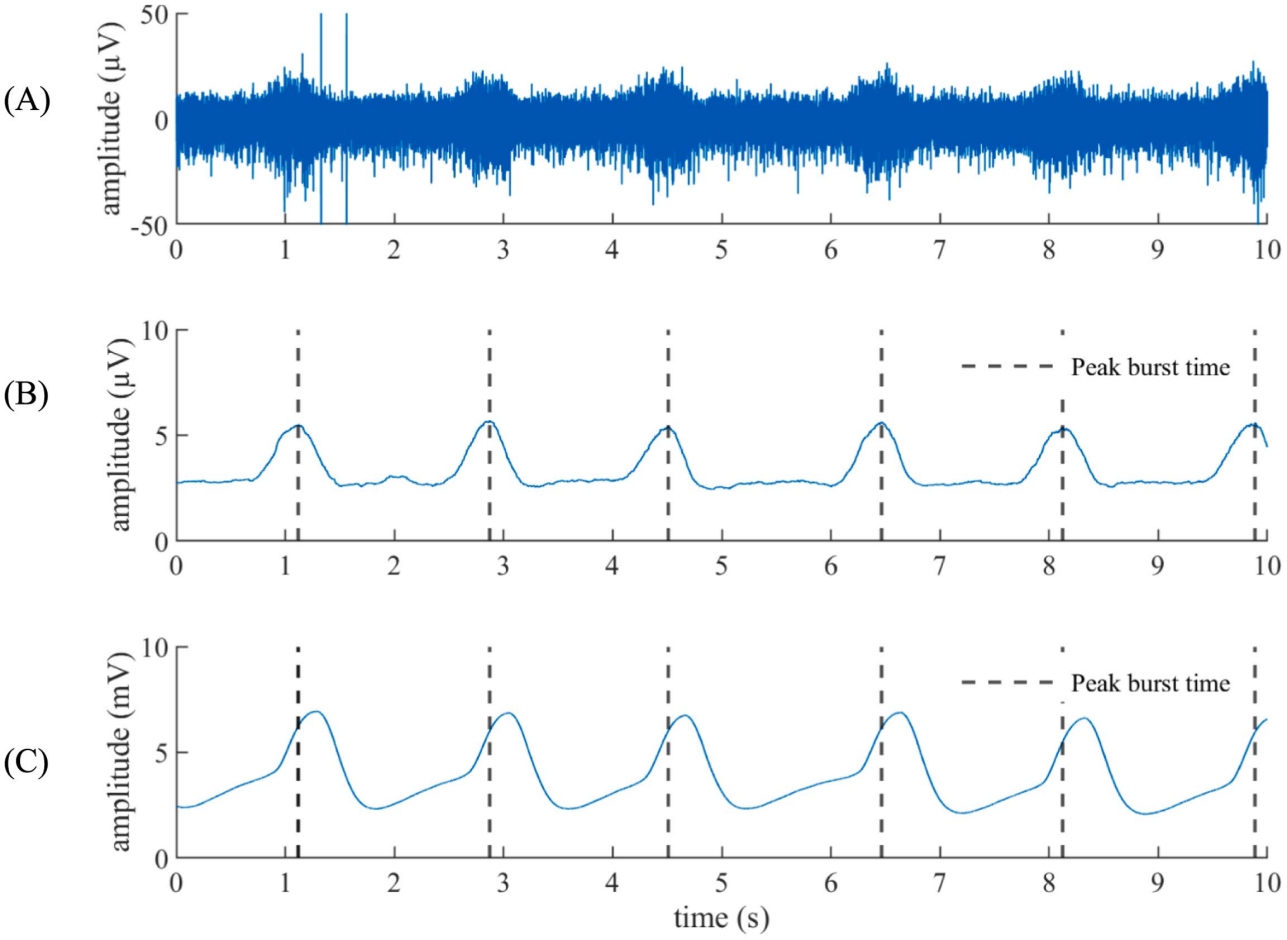
Respiration from the VENG versus the respiratory signal. **(A)** VENG signal. **(B)** Respiratory component extracted from the VENG. **(C)** Facemask-acquired respiration signal. The peak activity on the modulation burst time is remarked in black dashed lines, showing that the peak activity happened during the inhalation (when the respiratory signal goes up).

**Figure 2:**
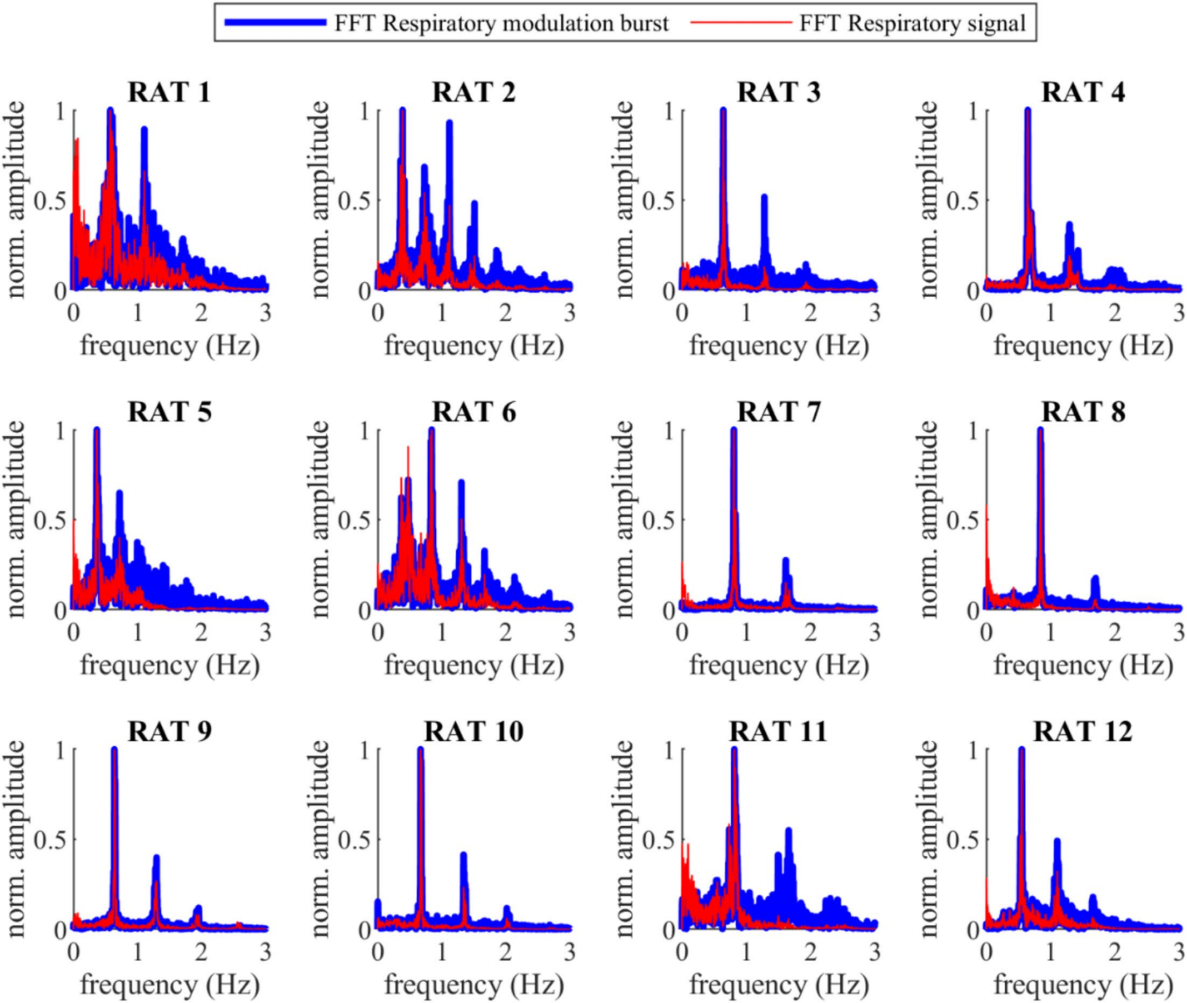
Fast Fourier Transform (FFT) of the VENG respiratory and the respiration signal during baseline for all 12 rats. The main prominence frequency peaks for both signals are perfectly aligned (the difference was lower than the frequency resolution of the FFT, 3.33 mHz).

Figure 1 compares VENG respiratory burst and respiration signal during baseline, showing that both have the same amplitude change pattern (illustration from rat N° 12). The peak of the respiratory bursts occurs during inspiration (when the respiratory signal voltage increases), which is consistent with the literature (Patros et al., 2022). The FFT of the respiratory bursts and the respiratory signal show that the dominant frequency values were identical for all rats (Figure 2), with no measurable deviation (any potential discrepancy under the FFT resolution of 3.33 mHz). Additionally, the PLI was 0.86 ± 0.08 (Refer to Supplementary Table 2). This confirms that the two signals were highly synchronous and that the vagus nerve maintained its functionality and integrity.

### 3.2 Changes in anesthesia level

**Figure 3:**
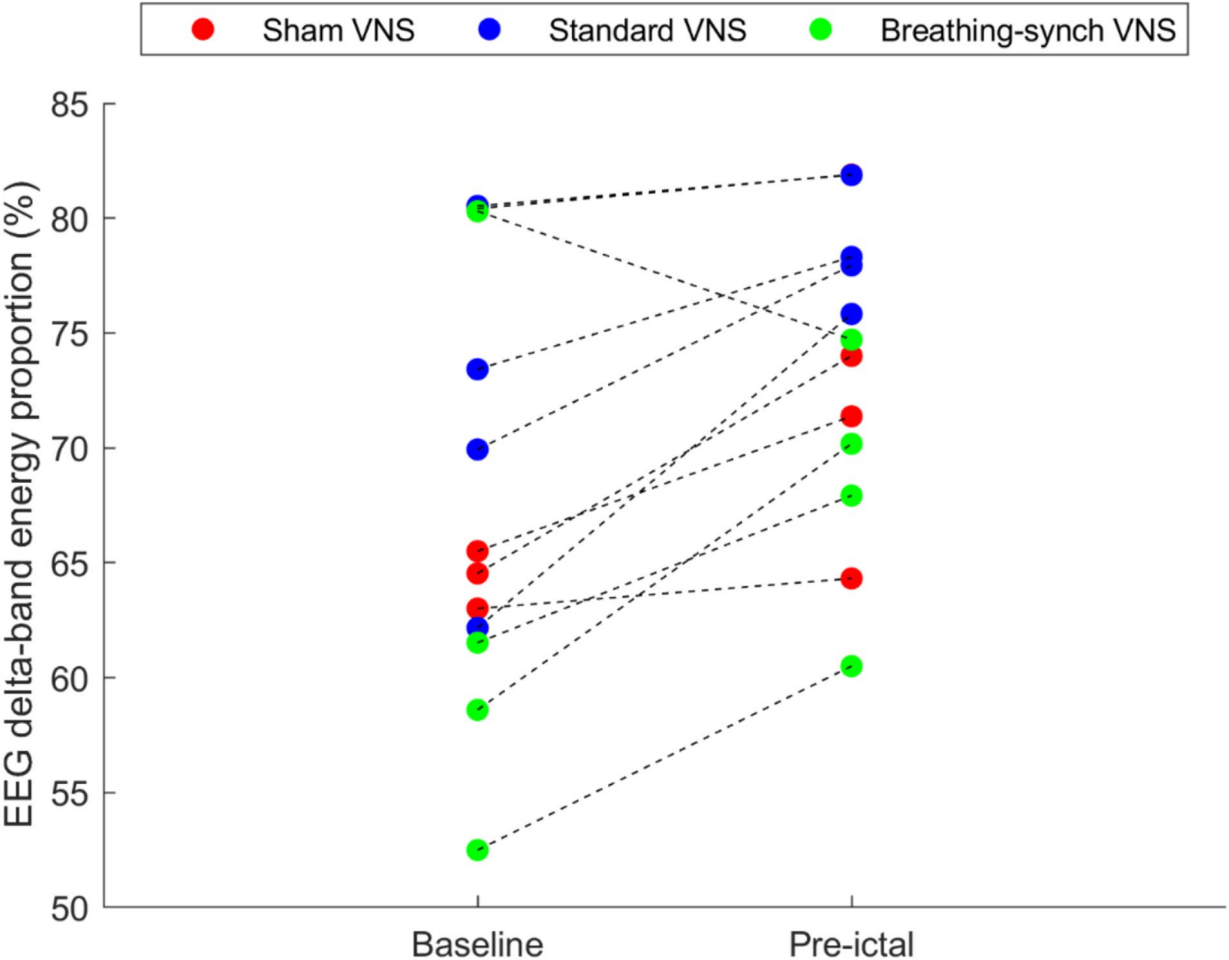
Average EEG delta-band energy proportion obtained during the baseline and during the pre-ictal period, per rat. Most of the rats (92%) experienced an increase in the EEG delta-wave energy proportion, and the remaining experienced a decrease (in the Breathing-synchronized VNS).

The baseline delta band energy proportion and the pre-ictal delta wave proportion were effectively obtained from the EEG signals. Figure 3 shows that 92% of the rats experienced increased EEG delta-band energy proportion in the preictal phase. The overall mean of delta wave proportions during baseline was 67.7 ± 9.3%, and 73.2 ± 6.7% for preictal, showing an increment of 5.5 ± 5.3%. The differences were normally distributed (Shapiro–Wilk test, *p*-value = 0.83 > 0.05), and the mean difference was statistically significant (Student’s *t*-test paired, *p*-value = 0.004 < 0.05), meaning an increase in the anesthesia level from baseline to preictal stages (Refer to Supplementary Table 3).

### 3.3 Effect of PTZ and VNS on the cardiorespiratory system

#### 3.3.1 Heart rate and breathing rate changes per group

##### 3.3.1.1 Sham VNS

**Figure 4:**
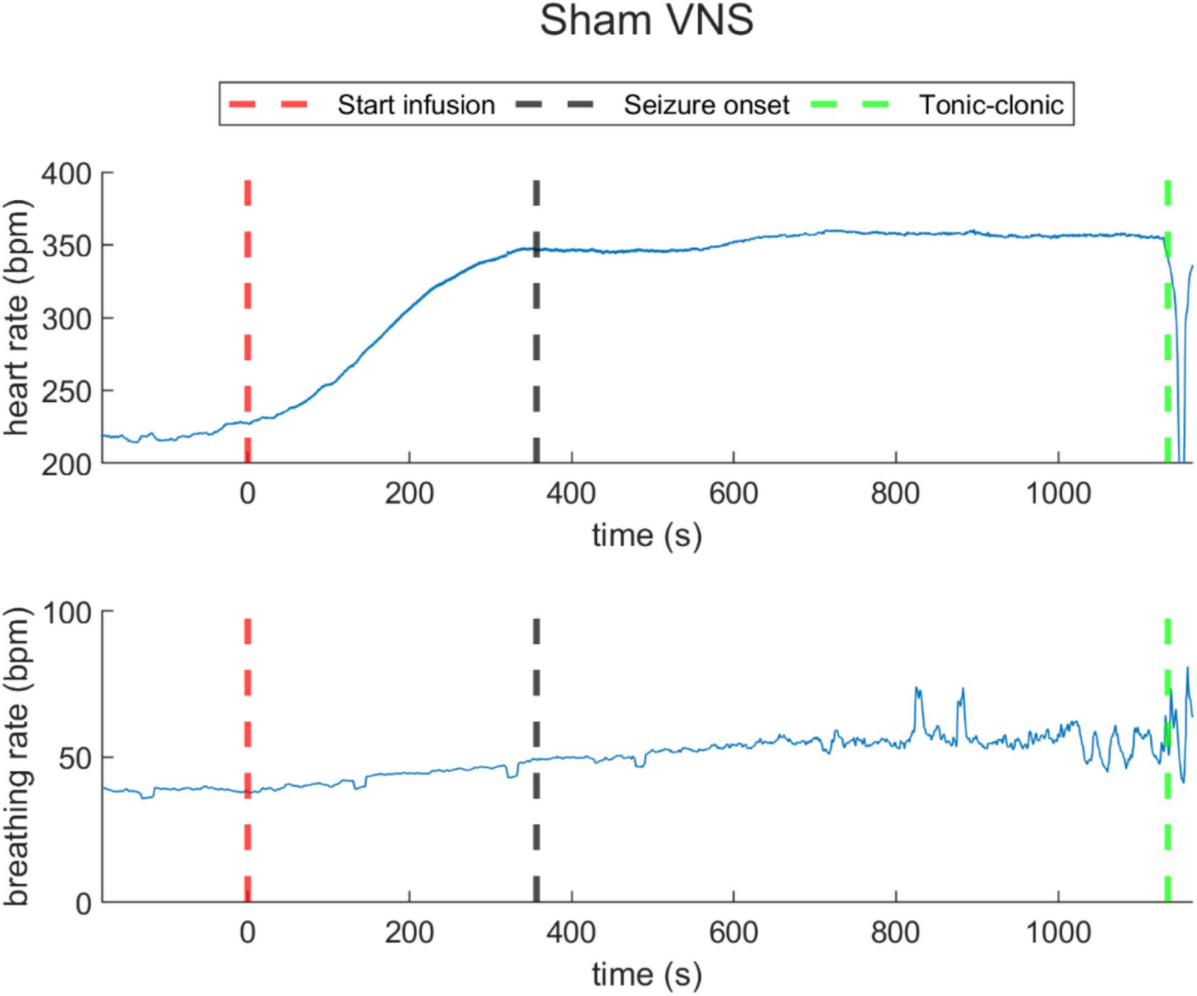
Heart rate (top) and breathing rate (bottom) evolution over time in a representative rat under sham VNS. The increasing of heart rate and respiratory rate was registered. The red dashed lines represent the starting time of infusion of PTZ (*t* = 0). The black dashed line represents the seizure onset and the green dashed line, the time when the rats reached tonic–clonic seizure and the infusion was stopped.

During sham stimulation, both heart and breathing rates increased compared to baseline levels (as illustrated in Figure 4 for rat N° 4, Refer to Supplementary Figures 1, 2 for details). During the last 30-s baseline, the average heart rate was 196.6 ± 22.5 bpm, and the average breathing rate was 32.8 ± 7.6 bpm. During the infusion, the rise in heart rate was consistent across all rats, showing an average increase of 79.2 ± 35.5 bpm with respect to baseline. For breathing rate, rats exhibited an average increase of 13.6 ± 1.8 bpm with respect to baseline. During the post-infusion period, the heart rate was 266.3 ± 45.1 bpm, and the breathing rate was 48.9 ± 4.1 bpm, both remaining above baseline levels, indicating an incomplete recovery (Refer to Supplementary Tables 4, 5 for details).

##### 3.3.1.2 Standard VNS

**Figure 5:**
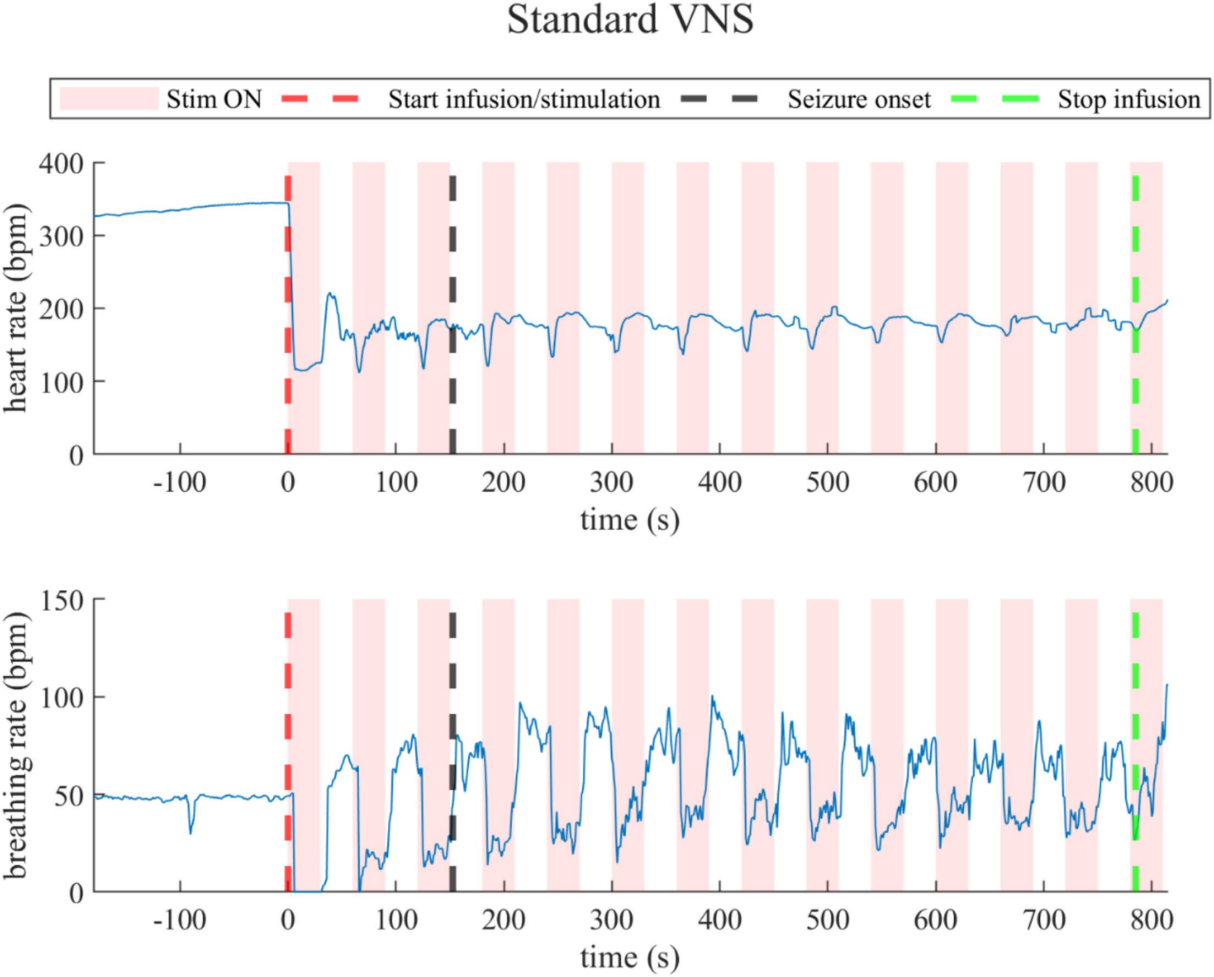
Heart rate (top) and breathing rate (bottom) evolution in the rat under standard VNS. At every stimulation onset, the heart rate drops quickly, but then recovers during the OFF periods, but did not return to baseline values. The breathing rate dropped during the ON stimulation times and increased during the OFF times.

Under standard VNS, both heart rate and breathing rate exhibited changes synchronous to VNS ON/OFF cycles, as illustrated in a representative rat (rat N° 7) in Figure 5 (Refer to Supplementary Figures 3, 4 for details). During the last 30-s baseline, the average heart rate was 297.5 ± 92.0 bpm, and the average breathing rate was 37.5 ± 14.6 bpm. During the overall stimulation/infusion time (including ON and OFF periods), all rats experienced a decrease in the heart rate of 120.0 ± 69.1 bpm, without returning to baseline values. During the ON periods, the heart rate was 171.6 ± 47.3 bpm, and during the OFF periods, 183.9 ± 48.3 bpm. Regarding breathing rate, during the stimulation/infusion time, there was an average increase of 13.0 ± 14.7 bpm. During the ON periods, there was an average breathing rate of 45.3 ± 7.6 bpm, and during the OFF periods, there was 55.8 ± 9.5 bpm. During the post-infusion period, due to the persistent VNS, the heart rate was 180.5 ± 56.4 bpm (still below baseline), and the breathing rate was 54.5 ± 3.2 bpm (still above baseline), indicating incomplete recovery (Refer to Supplementary Tables 6, 7 for details).

##### 3.3.1.3 Breathing-synchronized VNS

**Figure 6:**
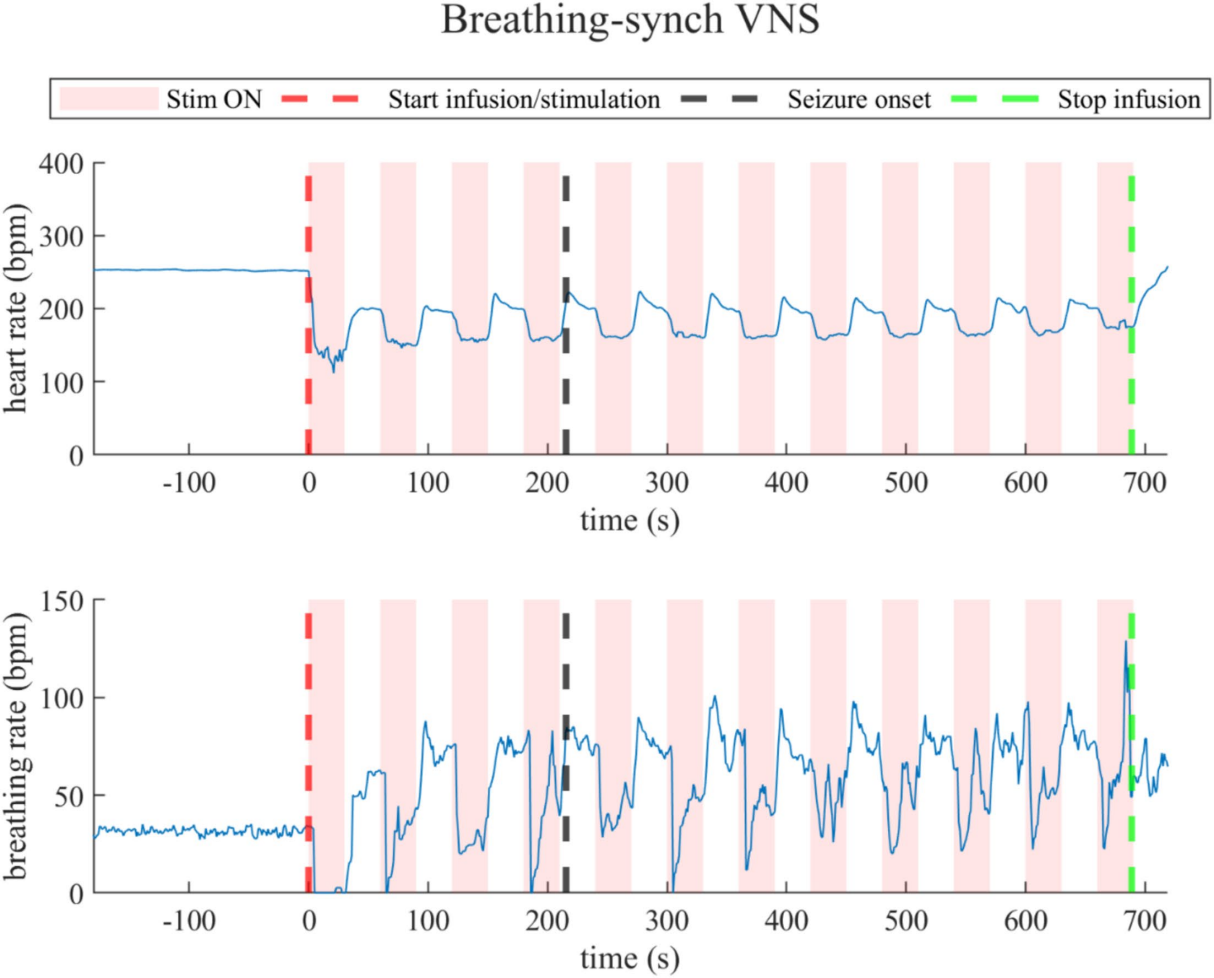
Heart rate (top) and breathing rate (bottom) evolution in rats under Breathing-synchronized VNS.

Similarly to standard VNS, breathing-synchronized VNS induced changes in heart rate and breathing rate, illustrated by the data of a representative rat (rat N° 10) in Figure 6 (Refer to Supplementary Figures 5, 6). During the last 30-s baseline, the average heart rate was 256.2 ± 71.2 bpm, and the average breathing rate was 39.1 ± 6.1 bpm. During the overall stimulation/infusion time (including ON and OFF periods), the heart rate had an average decrease of 84.9 ± 61.0 bpm, without returning to baseline values. During ON periods, the average heart rate dropped to 160.8 ± 31.6 bpm, and during OFF periods, as the standard VNS, there was a slight recovery of the heart rate to 182.5 ± 32.4 bpm, but still below the baseline values. Regarding breathing rate changes, there was an overall increase of 13.7 ± 10.4 bpm. During ON periods, the breathing rate was 45.7 ± 9.7 bpm; for OFF periods, the breathing rate was 61.3 ± 10.7 bpm. During the post-infusion period, the heart rate was 184.1 ± 38.7 bpm (still below baseline), and the breathing rate was 50.1 ± 9.7 bpm (still above baseline), indicating incomplete recovery due to the persistent VNS as for standard VNS (Refer to Supplementary Tables 8, 9).

#### 3.3.2 Comparison of heart rate changes between groups

**Figure 7:**
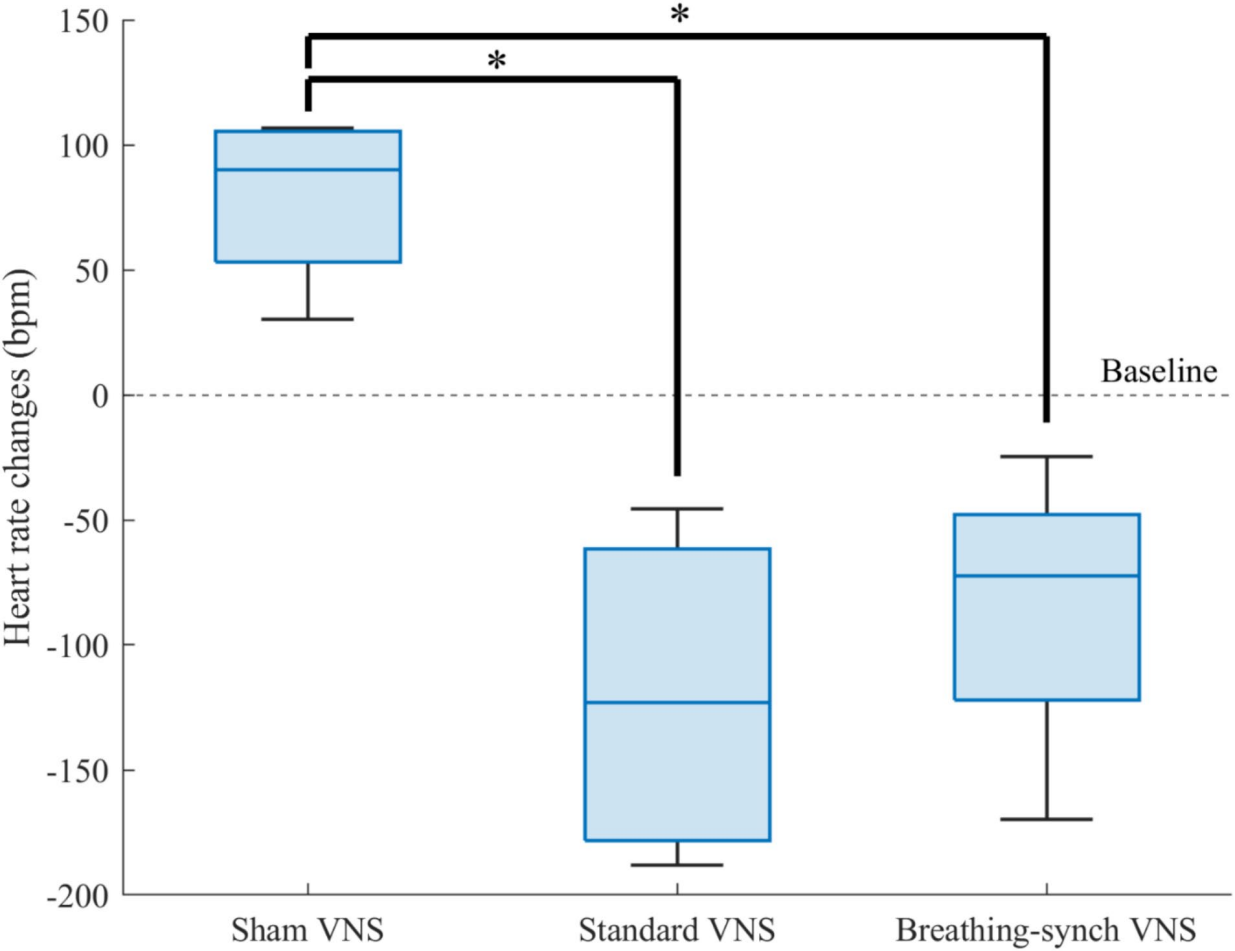
Heart rate changes respect to baseline for each group. The sham VNS heart rate changes was significantly higher than the standard VNS and breathing-synchronized VNS heart rate changes (*p value<0.05). There was no significant difference in the variance among groups.

The heart rate changes during the infusion period with respect to the baseline were compared for all the groups in Figure 7. The heart rate dropped below baseline values without recovery, except slightly in one rat in the groups under stimulation, while it increased in the sham group. All the heart rate changes distribution among groups were normally distributed (Shapiro–Wilk test, *p*-values of 0.28, 0.40, and 0.44 for sham, standard VNS, and breathing-synchronized VNS, respectively).

Compared to sham heart rate changes (+79.2 ± 35.5 bpm), there was a significant difference in the stimulation groups, standard VNS (−120.0 ± 69.1 bpm; Student’s *t*-test, corrected *p*-value = 0.0065 < 0.05) and breathing-synchronized VNS (−84.9 ± 61.0 bpm; Student’s *t*-test, corrected *p*-value = 0.0105 < 0.05). There was no significant difference between VNS groups (Student’s *t*-test, corrected *p*-value = 1.0 > 0.05). The variance among groups was not significantly different (*F*-test, all corrected *p*-values = 1.0 > 0.05). Effect size calculations showed a large difference between the sham VNS and standard VNS (Cohen’s *d* = 3.1), as well as between the sham VNS and breathing-synchronized VNS (Cohen’s *d* = 2.9). The effect size between the standard VNS and breathing-synchronized VNS showed a medium difference (Cohen’s *d* = 0.5). This indicates that both stimulations caused a significant constant drop in the heart rate.

#### 3.3.3 Comparison of breathing rate changes between groups

**Figure 8:**
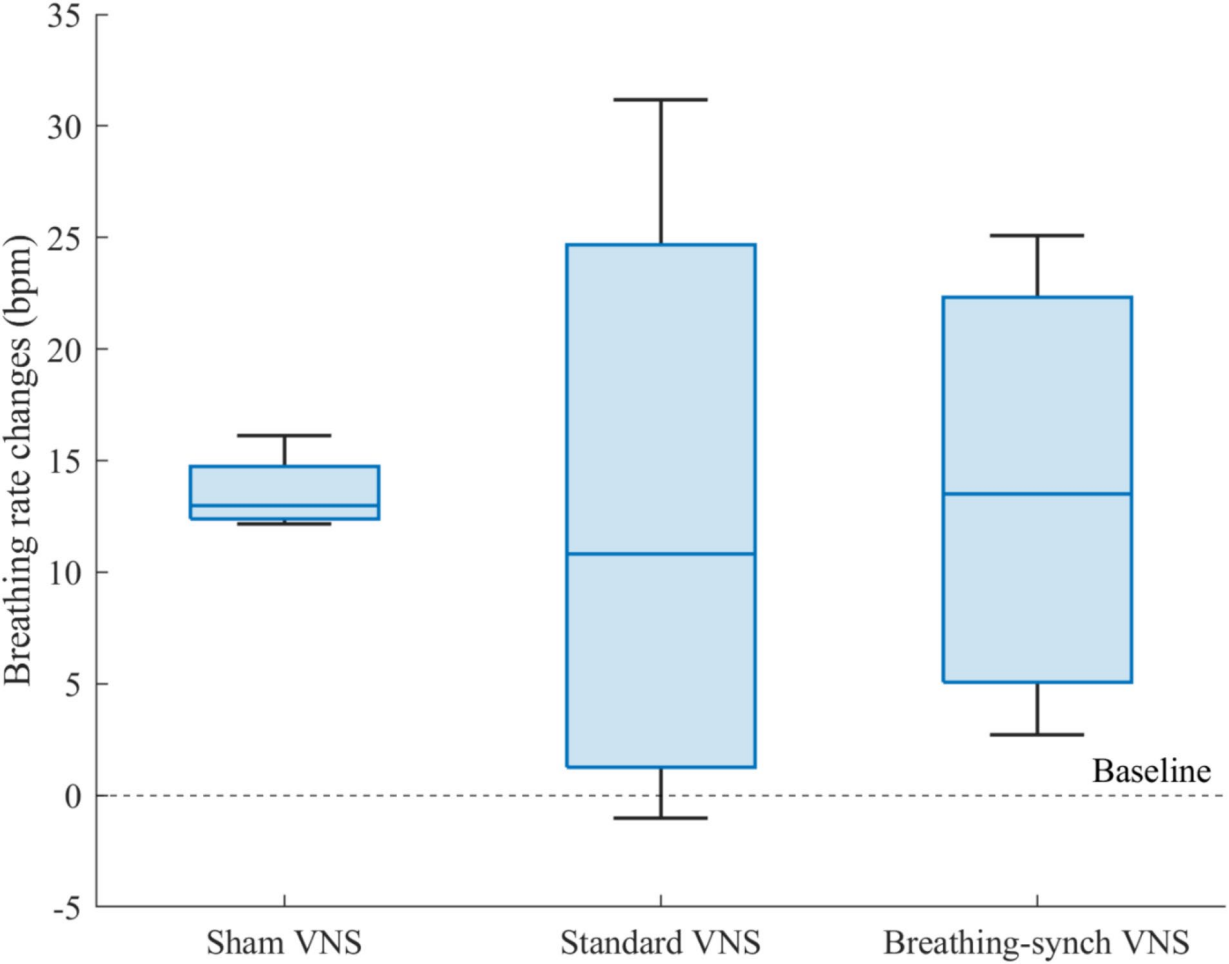
Breathing rate changes respect to baseline for each group. There was no significant mean difference among groups.

Figure 8 shows the distribution of breathing rate changes among groups. The average increase presented in the sham VNS (13.6 ± 1.8 bpm), standard VNS (13.0 ± 14.7 bpm) and breathing-synchronized VNS (13.7 ± 10.4 bpm) were normally distributed (Shapiro–Wilk test, *p*-values of 0.24, 0.64, and 0.62 for sham, standard VNS, and breathing-synchronized VNS, respectively).

There was no significant mean difference among groups (Student’s *t*-test, all corrected *p*-values 1.0 > 0.05). Effect size indicated no meaningful difference between the sham VNS and standard VNS (Cohen’s *d* = 0.0), as well as between the sham VNS and breathing-synchronized VNS (Cohen’s *d* = 0.0). The effect size between the standard VNS and breathing-synchronized VNS was not meaningful either (Cohen’s *d* = 0.1). However, there was a significant difference in the variance between the sham group and the standard VNS group (*F*-test, corrected *p*-value = 0.018 < 0.05) and the breathing-synchronized VNS (*F*-test, corrected *p*-value = 0.049 < 0.05). There was no significant variance difference between the stimulation groups (*F*-test, corrected *p*-value = 1.0 > 0.05). While the sham VNS group exhibited relatively consistent respiratory changes, the stimulation groups showed a wide range of responses, with some rats having a noticeable increase in breathing rate compared to baseline, while others had only a slight change compared to baseline. The substantial variance in the stimulated group could be attributed to both the ON/OFF variations and the drifting breathing patterns observed during stimulation.

#### 3.3.4 Apneas

**Figure 9:**
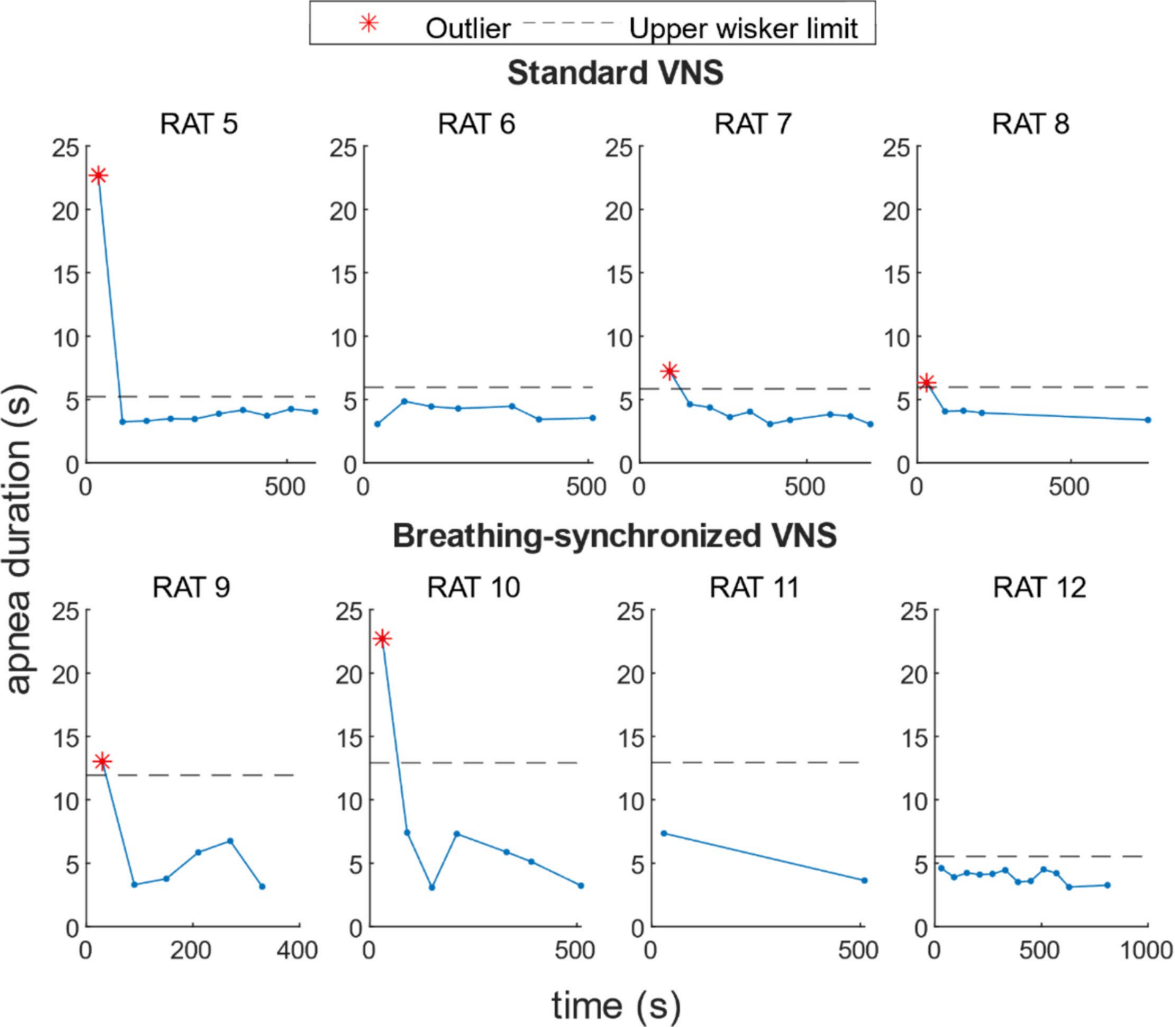
Apnea duration over time per rat under VNS. For 50% of the rats, there was a higher apnea duration during the first stimulation onset, and for 12% of the rats, during the second stimulation onset.

For standard VNS, the mean maximal apnea duration during stimulation for rats N° 5, N° 6, N° 7, and N° 8 was 5.6 ± 6.0 s, 4.0 ± 0.7 s, 4.1 ± 1.2 s, and 4.4 ± 1.1 s, respectively. For breathing-synchronized VNS, the mean maximal apnea duration during stimulation for rats N° 9, N° 10, N° 11, and N° 12 was 6.0 ± 3.8 s, 7.8 ± 6.8 s, 5.5 ± 2.6 s, and 4.0 ± 0.5 s, respectively (Refer to Supplementary Table 10 for details). Figure 9 shows the apnea duration over time per rat. In 62% of the rats under VNS, the first apnea duration was much longer than subsequent apnea durations (outliers), that could indicate an initial stress response followed by an adaptation.

#### 3.3.5 VNS modulation on heart rate and breathing rate

**Figure 10:**
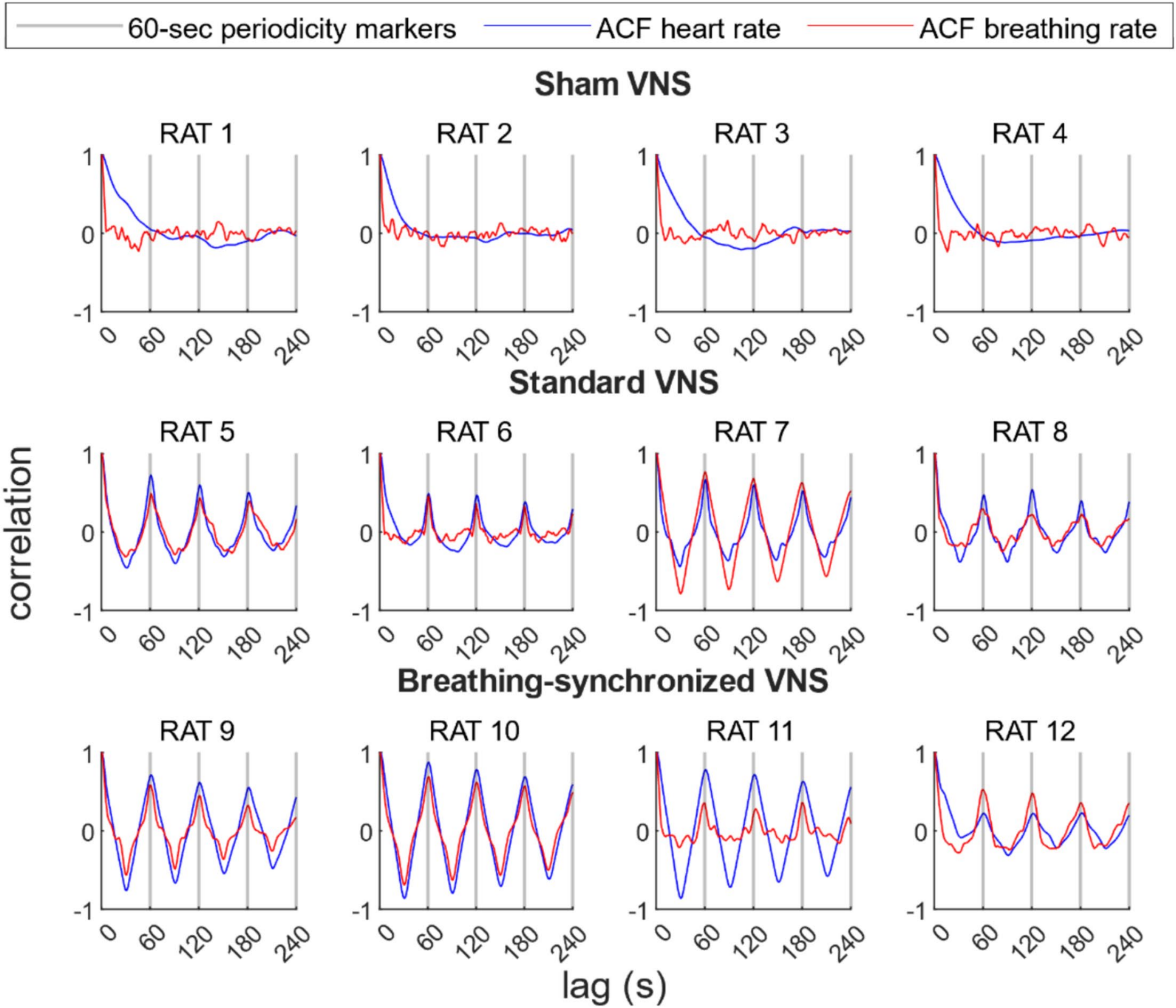
Autocorrelation function (ACF) of the heart rate and breathing rate per rat. For the sham group, there were no prominent peaks every 60 s. For the standard VNS and breathing-synchronized VNS, prominent and statistically significant peaks were located every 60-s lag, representing 60-s periodicity, confirming the stimulation modulation.

The autocorrelation analysis revealed a significant periodicity in both heart and breathing rates in the VNS groups, with a prominent peak correlation, around every 60 s, matching the stimulation period. Figure 10 shows the ACF for each rat’s heart rate and breathing rate. In the sham VNS group, no prominent peaks at 60-s intervals were observed in either heart or breathing rate, confirming the expected lack of periodicity. In contrast, both the standard VNS and breathing-synchronized VNS groups show a pronounced and statistically significant correlation peaks at 60-s intervals, confirming their periodicity and, therefore, the periodical influence of stimulation on heart and breathing rates.

The heart rate variability was 46.1 ± 24.0 ms in the standard VNS group and 59.4 ± 39.3 ms in the breathing-synchronized VNS group (Refer to Supplementary Table 11), with variability being normally distributed in both groups (Shapiro–Wilk test, *p*-values of 0.83 and 0.59, respectively). There was no significant difference between the standard and breathing-synchronized VNS groups (Student’s *t*-test, *p*-value = 0.58 > 0.05). The effect size between the standard VNS and breathing-synchronized VNS showed a small difference (Cohen’s *d* = 0.4). The breathing rate variability was 1064.0 ± 556.2 ms in the standard VNS group and 732.9 ± 269.0 ms in the breathing-synchronized VNS group, with both groups showing normal distributions (Shapiro–Wilk test, *p*-values of 0.41 and 0.33, respectively). No significant difference was observed between the standard and breathing-synchronized VNS groups (Student’s *t*-test, *p*-value = 0.32 > 0.05). The effect size between the standard VNS and breathing-synchronized VNS showed a moderate difference (Cohen’s *d* = 0.7). These findings indicate that VNS modulation strength was similar between the standard VNS and breathing-synchronized VNS.

### 3.4 Seizure susceptibility

**Figure 11:**
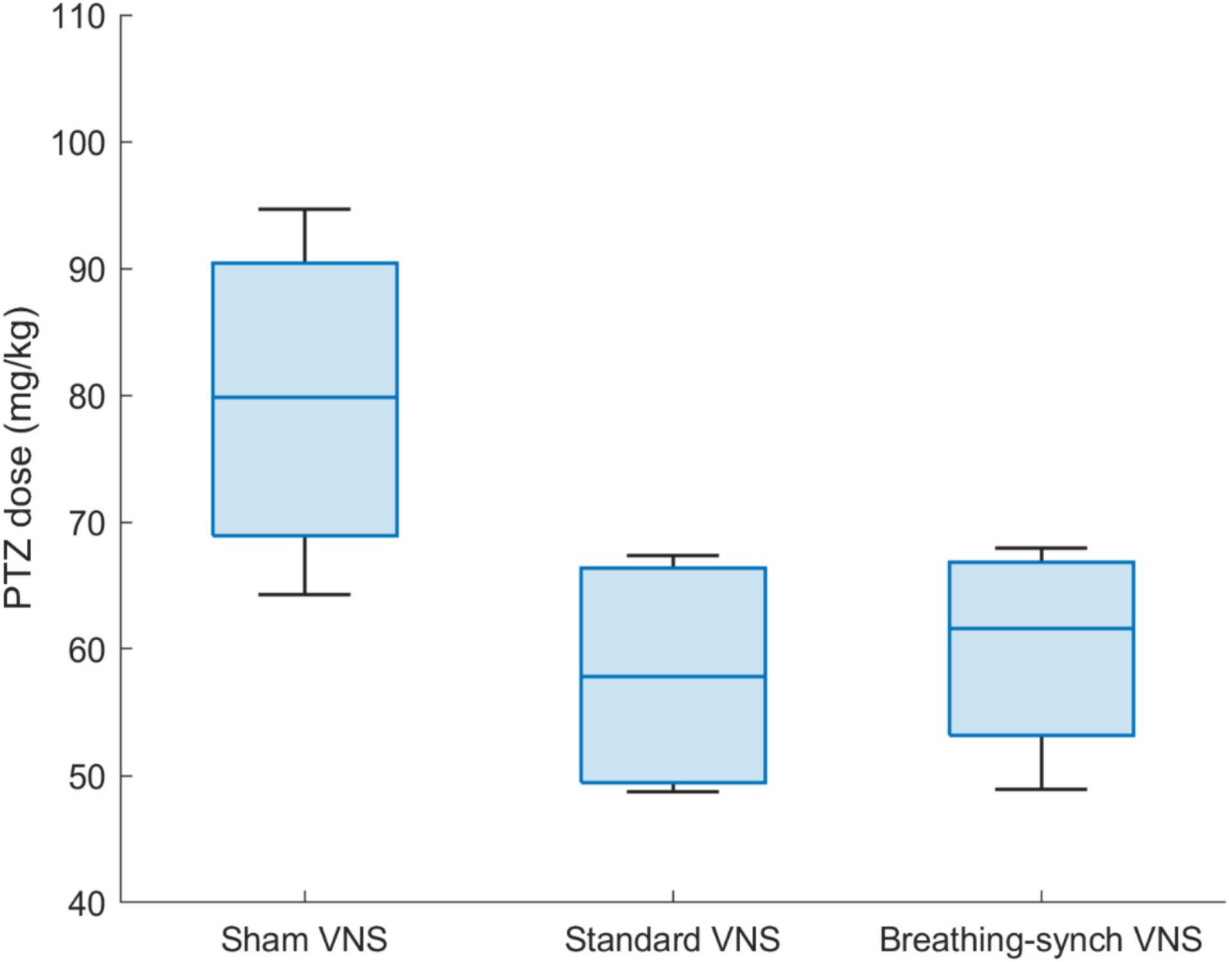
PTZ dose delivered by each group of rats to reach tonic–clonic stage. The mean dose delivered in the sham VNS group is significantly higher than the Standard VNS and Breathing-synchronized VNS (*p-value<0.05).

The PTZ threshold was evaluated on the basis of the total dose infused at the time the tonic–clonic stage was reached. The PTZ threshold was normally distributed in all groups (Shapiro Wilk test, *p*-values of 0.87, 0.13, and 0.57 for sham, standard VNS, and breathing-synchronized VNS, respectively, Refer to Supplementary Table 12). Figure 11 shows the PTZ dosage delivered by each group. The mean PTZ dose delivered in the sham VNS group (79.7 ± 13.4 mg/kg) was higher but without reaching statistical significance compared to the standard VNS (57.9 ± 9.8 mg/kg; Student’s *t*-test, corrected *p*-value = 0.12 > 0.05), and compared to the breathing-synchronized VNS (60.0 ± 8.7 mg/kg; Student’s *t*-test, corrected *p*-value = 0.15 > 0.05), and there was no statistically significant difference between the standard VNS and the breathing-synchronized VNS (Student’s *t*-test, corrected *p*-value = 1.0 > 0.05). However, there was a large effect size between the sham VNS and standard VNS (Cohen’s *d* = 1.6), as well as between the sham VNS and breathing-synchronized VNS (Cohen’s *d* = 1.5), indicating a meaningful difference. The effect size between the standard VNS and breathing-synchronized VNS was small (Cohen’s *d* = 0.2). These could suggest that even without showing statistical significance, the PTZ threshold is decreased by using stimulation. Therefore, the stimulation increased the seizure susceptibility.

The mean seizure latency was 291.5 ± 84.4 s for sham VNS, 200.5 ± 59.5 s for standard VNS, and 206.9 ± 66.0 s for breathing-synchronized VNS (Refer to Supplementary Table 13), both normally distributed (Shapiro–Wilk test, *p*-values of 0.27, 0.33 and 0.55 respectively). No significant differences were found between sham VNS and standard VNS (Student’s *t*-test, corrected *p*-value = 0.39 > 0.05) nor between sham VNS and breathing-synchronized VNS (Student’s *t*-test, corrected *p*-value = 0.50 > 0.05). Similarly, there was no significant difference between standard VNS and breathing-synchronized VNS (Student’s *t*-test, corrected *p*-value = 1.0). However, there was a large effect size between the sham VNS and standard VNS (Cohen’s *d* = 1.1), as well as between the sham VNS and breathing-synchronized VNS (Cohen’s *d* = 1.0), indicating a meaningful difference in latency. The effect size between the standard VNS and breathing-synchronized VNS was small (Cohen’s *d* = 0.1). This shows that seizures occurred earlier under VNS than those in the sham VNS.

The mean ictal seizure duration was 664.4 ± 126.4 s for sham VNS, 494.3 ± 149.7 s for standard VNS, and 513.3 ± 50.9 s for breathing-synchronized VNS (Refer to Supplementary Table 13), being all normally distributed (Shapiro–Wilk test, *p*-values of 0.38, 0.57 and 0.33 respectively). No significant differences were observed between sham VNS and standard VNS (Student’s *t*-test, corrected *p*-value = 0.40 > 0.05), as well as between sham VNS and breathing-synchronized VNS (Student’s *t*-test, corrected *p*-value = 0.21 > 0.05). The difference between standard VNS and breathing-synchronized VNS did not reach statistical significance either (Student’s *t*-test, corrected *p*-value = 1.0 > 0.05). Despite this, there was a large effect size between the sham VNS and standard VNS (Cohen’s *d* = 1.1), as well as between the sham VNS and breathing-synchronized VNS (Cohen’s *d* = 1.4), indicating a meaningful difference in the ictal duration. The effect size between the standard VNS and breathing-synchronized VNS was small (Cohen’s *d* = 0.1), indicating that the ictal seizure duration differences were not meaningful. This shows that severe seizures (tonic–clonic) are reached before under stimulation.

The mean post-ictal seizure duration was 63.9 ± 33.8 s for sham VNS (normally distributed, Shapiro–Wilk test, *p*-value = 0.32), 66.7 ± 31.0 s for standard VNS (not normally distributed, Shapiro Wilk test, *p*-value = 0.040 < 0.05), and 58.8 ± 24.2 s for breathing-synchronized VNS (normally distributed, Shapiro Wilk-test, *p*-value = 0.55) (Refer to Supplementary Table 13). No significant differences were observed between sham VNS and standard VNS (Wilcoxon test, corrected *p*-value = 1.0 > 0.05), as well as between sham VNS and breathing-synchronized VNS (Student’s *t*-test, corrected *p*-value = 1.0 > 0.05). The difference between standard VNS and breathing-synchronized VNS did not reach statistical significance either (Wilcoxon test, corrected *p*-value = 1.0 > 0.05). There was a small size effect between the sham VNS and standard VNS (Cohen’s *d* = 0.1), as well as between the sham VNS and breathing-synchronized VNS (Cohen’s *d* = 0.2). The effect size between the standard VNS and breathing-synchronized VNS was also small (Cohen’s *d* = 0.2). This shows that the post-ictal seizure duration did not change under stimulation compared to sham VNS.

#### 3.4.1 Influence of weight and age on seizure susceptibility

To further assess whether differences in age or weight could have influenced the results, we compared these variables across the experimental groups. The ages and weight of the rats per group were 2.8 ± 0.4 months and 361.3 ± 14.4 grams for the sham VNS, 3.0 ± 0.6 months and 340.0 ± 39.2 grams for the standard VNS group, and 3.5 ± 1.2 months and 365.0 ± 14.7 grams for the breathing-synchronized VNS group. The ages of the rats per group were normally distributed (Shapiro–Wilk test, *p*-values of 0.094, 0.64, and 0.27, respectively) as well as the weight (Shapiro–Wilk test, *p*-values of 0.27, 0.85, and 0.35, respectively). The variances presented in weight and age were not statistically significant. There were no statistical differences in weight (Student’s *t*-test, corrected *p*-values of 1.0, 1.0, and 0.83 > 0.05) and age (Student’s *t*-test, all corrected *p*-values = 1.0 > 0.05) among the groups.

The PTZ threshold has no significant linear relationship with the weight of the rats (*R*^2^ = 0.046, *p*-value 0.50 > 0.05). It has a significant linear relationship with the age (*p*-value 0.0274 < 0.05), but with a moderate fit (*R*^2^ = 0.40). This suggests that weight can be a factor that explains the PTZ threshold but is not highly predictive (Refer to Supplementary Figures 7, 8).

#### 3.4.2 Influence of heart rate and breathing rate VNS modulation on seizure susceptibility

The PTZ threshold showed a significant but moderate linear relationship with heart rate variability (*R*^2^ = 0.38, *p*-value = 0.032), but no significant relationship with breathing rate variability (*R*^2^ = 0.21, *p*-value = 0.12). Furthermore, the PTZ threshold showed a moderate but non-significant linear relationship with the combined heart rate and breathing rate variability (*R*^2^ = 0.43, *p*-value = 0.083) (Refer to Supplementary Figures 9–11).

#### 3.4.3 Influence of heart rate and breathing rate baseline values on seizure susceptibility

The PTZ threshold did not present a significant linear relationship with the heart rate baseline values nor breathing rate baseline values (*R*^2^ = 0.005 and 0.05; *p*-value of 0.82 > 0.05 and 0.47 > 0.05, respectively), neither combined (*R*^2^ = 0.11; *p*-value = 0.60 > 0.05), showing no significant correlation (Refer to Supplementary Figures 12–14).

#### 3.4.4 Influence of anesthesia level on seizure susceptibility

The PTZ threshold did not present a significant linear relationship with the EEG delta-band energy proportion (*R*^2^ = 0.0001; *p*-value = 0.97 > 0.05), showing a weak correlation (Refer to Supplementary Figure 15).

#### 3.4.5 Influence of mean heart rate and breathing rate changes on seizure susceptibility

**Figure 12:**
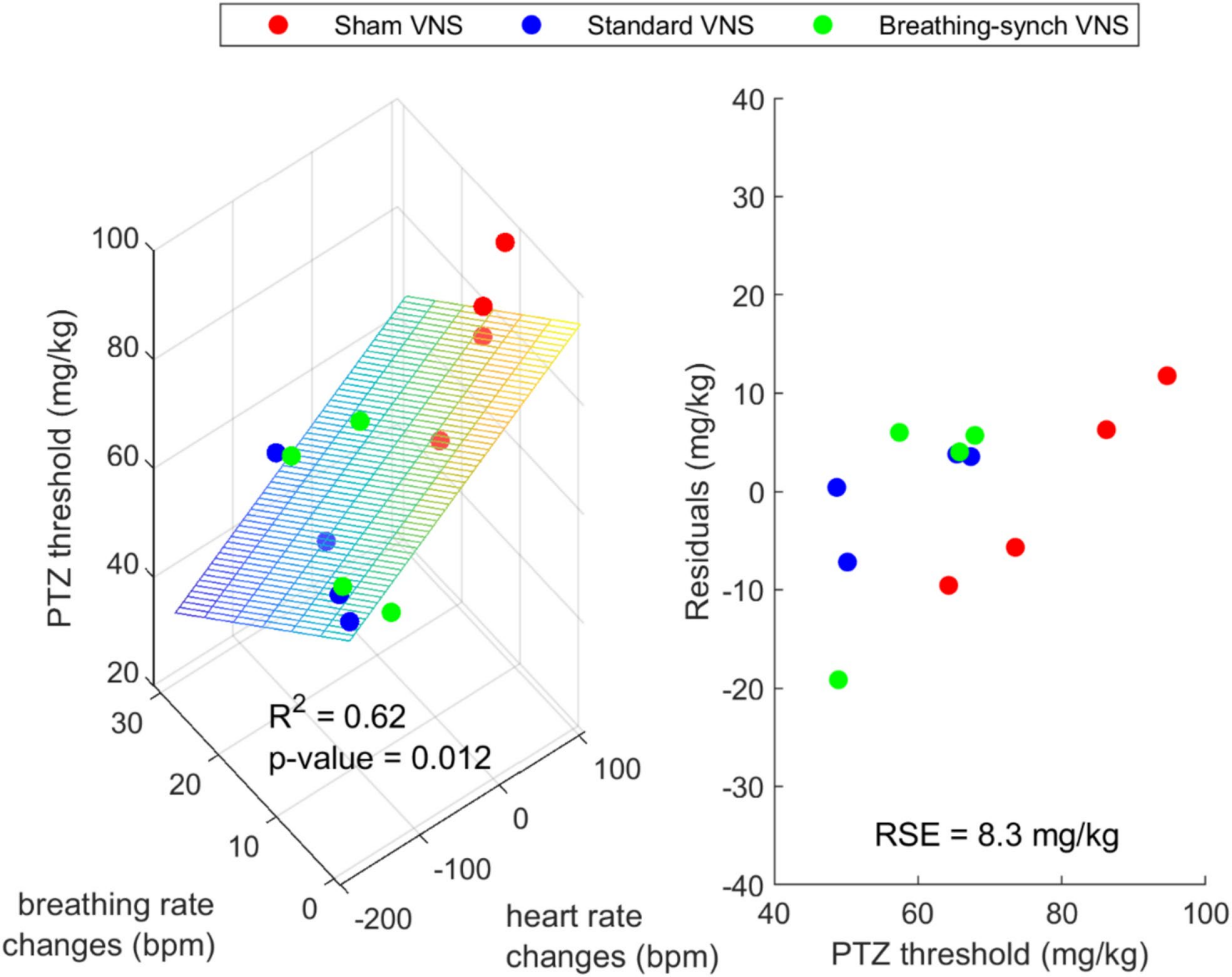
Left: 3D scatter plot with fitted plane showing a possible linear relationship between the PTZ dose delivered, the average heart rate changes and the average breathing rate changes. Right: Residuals of the regression (RSE = Residual Standard Error).

The linear relationship between the PTZ threshold and the average heart rate and average respiratory rate changes was not significant (*R*^2^ = 0.28 and 0.09; *p*-value 0.075 > 0.05 and 0.34 > 0.05, respectively) (Refer to Supplementary Figures 16, 17). However, the PTZ threshold has a significant linear relationship to the combined average heart rate changes and average breathing rate changes (*R*^2^ = 0.62, *p*-value 0.012 < 0.05). Figure 12 shows a 3D scatter plot linking the PTZ dose delivered, the average heart rate changes, and the average breathing rate changes. This suggests that the mean heart rate and breathing rate changes explain the majority of the variation in the PTZ threshold; while not perfect or very strong, it represents the best relationship found.

#### 3.4.6 Inter-rat variability

Inter-rat variability was assessed on baseline parameters: anesthesia level, age, weight, breathing rate, and heart rate using the IQR. One outlier was found in the weights (300 g, in rat N°8, under standard VNS) and in the ages (5.23 months, rat N°9, under breathing-synchronized VNS). Given that the PTZ i.v. administration was normalized by weight, the outlier in weight was unlikely to be a confounding factor to determine the PTZ threshold. While the rat ages presented one outlier, its corresponding PTZ threshold (48.94 mg/kg) fell within the IQR range of its group (38.77–82.85 mg/kg), indicating that it is not considered an extreme outlier within its stimulation group. This suggests that these deviations are unlikely to have a meaningful impact on the observed differences in the PTZ threshold.

## 4 Discussion

This work explores the impact of VNS on the autonomic nervous system in anesthetized rats under i.v. PTZ infusion, comparing the effects of 3 types of stimulation: sham, standard and breathing-synchronized VNS. During sham stimulation, both heart and breathing rates increased compared to baseline levels caused by the PTZ, consistent with previous studies (Stumpp et al., 2020). Under stimulation, the rats experienced variations in both heart and respiration rates. A significant average drop in the heart rate was observed in the stimulation groups. Although the average breathing rate increased similarly to the sham groups, the variation in breathing rate was significantly higher in the stimulation groups.

Unlike our expectations, breathing-synchronized VNS did not mitigate the cardiorespiratory side effect, as no significant differences were found in the heart and breathing rate variability, and mean heart rate and breathing rate changes compared to the standard VNS. In that regard, intermittent stimulation (i.e., applying stimulation during the exhalation) for 30 s has the same effect as continuously stimulating for 30 s as long as the total number of stimulation pulses is equal in both cases. A possible explanation is that the VNS cumulatively impacts the cardiorespiratory system (temporal integration). Future research must be done to confirm this hypothesis.

The observed drop in the heart rate is consistent with previous literature (McLachlan, 1993; Woodbury and Woodbury, 1990), in keeping with the parasympathetic innervation of the heart via the vagus nerve, where stimulation reduces heart rate and cardiac contractility (Yao et al., 2023). However, unlike previous reports (Woodbury and Woodbury, 1990; McLachlan, 1993; Ahmed et al., 2020), the heart rate in most rats did not return to baseline values during the OFF periods of stimulation. This sustained decrease could be attributed to prolonged vagal activation and continued stimulation of baroreceptor afferents, preventing the heart rate from returning to baseline values. This could also explain the uniform drop in the heart rate among the groups of rats, since the variability in the changes of heart rate observed across all groups was not significantly modified.

Regarding the respiration, under VNS, the presence of hypopnea and the possibility for apneas during prolonged stimulation trains has been confirmed in previous studies (McLachlan, 1993; Woodbury and Woodbury, 1990). Therefore, a decrease in respiratory rate was anticipated in the groups subjected to standard VNS. The vagus nerve afferent fibers control the respiration mechanically driven by the abdominal and phrenic nerves (Molkov et al., 2014). The VNS affects the breathing patterns. Although apneas were observed during the ON period of VNS, the average breathing rate in the stimulation groups was not significantly different from that of the sham group, showing a similar increase. In contrast, the variance in breathing rate changes was significantly different, with the stimulation groups exhibiting the largest variations compared to the sham group. This discrepancy can be explained by the fact that some rats experienced a drop in breathing rate apnea, followed by a slow recovery, while others showed a rapid increase in breathing rate during stimulation.

The PTZ dose required to induce tonic–clonic seizures was higher in the sham VNS group compared to the stimulation groups, and seizures also occurred earlier under VNS. Although the results were not statistically significant after correction, the trend remains evident and meaningful, indicated by the effect size. The impact of VNS on the autonomic system is the most predominant variable in our experiments that could explain this. However, the age and weight of the rats, as well as the use of ketamine, were also considered. Regarding the age of the rat, Klioueva et al. (2001) described that older rats are more sensitive to PTZ. However, they do not report a significant difference in the PTZ threshold dose in Wistar rats older than 90 days. In our study, there were no significant age differences between the groups. In addition, the *R*^2^ value obtained (0.40) suggests that age is insufficient to explain the different seizure susceptibility. The weight of the rat was discarded as a factor that could influence seizure susceptibility since the dosage was normalized by the weight. It is also possible that the anesthetic/antiepileptic effect of the ketamine (Fujikawa, 2019) was decreasing, making the rat more susceptible to PTZ. However, in 87% of the stimulated rats, the EEG delta-band energy proportions increased. In that regard, the rats did not regain consciousness, even under VNS. Additionally, the baseline state of heart rate, breathing rate, and anesthesia levels were not able to explain the differences in the PTZ threshold.

Regarding the cardiorespiratory VNS effects’ link to the PTZ threshold, heart rate variability showed a significant but moderate linear relation with the PTZ threshold. However, there was a strong link between heart rate changes, breathing rate changes, and the PTZ threshold. This suggests that both cardiovascular and respiratory effects of VNS may play a role in modulating seizure susceptibility. One possible explanation for this strong link is the impact of VNS on blood pressure and oxygenation levels. Stimulating the vagus nerve causes drops in blood pressure levels (Yetis et al., 2022). Hence, it is likely that the sustained bradycardia we observed in this study was also accompanied by an induced sustained low blood pressure in most of our rats under VNS, causing hypotension. The main limitation of this assumption is that blood pressure was not measured, and it was not possible to observe the compensatory mechanism for blood pressure. However, heart rate and blood pressure are linked, so it is reasonable to think there was a constant low blood pressure. Regarding the respiratory changes, despite the limitation of not measuring oxygen saturation, it is reasonable to infer the presence of respiratory hypoxia based on apnea duration. In 62% of the rats under VNS, the apnea duration rapidly decreased, suggesting an adaptation to VNS that may be linked to the hypoxia chemical feedback mechanism (Molkov et al., 2014). Zaaimi et al. (2005) describe the three phases of respiration during VNS (in 10 children in a sleep state). Initially, there is a phase of apnea or rapid, shallow breathing, leading to hypoxia. Secondly, there is a chemoreceptor excitation due to the generated hypoxia prompting a progressive return of respiration through hypoxia-induced pulmonary stretch receptor and chemoreceptor excitation. In the third phase, when VNS is stopped, respiration may remain enhanced due to the persistent hypoxic condition. A similar mechanism may explain the observed adaptation in the rats under VNS. The persistent hypoxic condition could drive the initial rapid decrease in apnea duration, followed by its stabilization at shorter durations.

The drop in the heart rate could cause hypotension, and the presence of apnea could cause respiratory hypoxia, which we hypothesized could contribute to an increased seizure susceptibility in anesthetized conditions. Hypoxia and hypotension can subsequently lead to cerebral hypoxia, which disrupts normal brain function. Cerebral hypoxia induces neuronal inflammation (Chiavegato et al., 2014) and alters the balance of brain excitability (Sanz and Garcia-Gimeno, 2020; Vezzani et al., 2011; Petroff, 2002) by affecting neurotransmitter systems and neuronal activity. The rationale is that impaired oxygen delivery to the brain, coupled with the resulting neuroinflammation and changes in excitability, could lower the PTZ threshold for seizure activity. Previous studies discussed the change of excitability balance in the brain under hypoxia/hypotension, showing glutamate and GABA changes. Phillis and Walter (1989) described that hypoxia/hypotension has an acute impact, increasing the glutamate release after 5 min of hypoxia. Kubo et al. (1990) also mention that chemical induced-hypotension decreases the level of GABA in brain regions such as the rostra1 ventrolateral medulla (RVL), caudal ventrolateral medulla (CVL), and NTS regions of the rat. The glutamate-GABA acute imbalance caused by hypoxia/hypotension could explain our results of VNS-induced seizure susceptibility.

These findings highlight the importance of optimizing stimulation parameters to avoid adverse effects due to overstimulation. However, this study has limitations. The absence of oxygen saturation and blood pressure measurements restricts the interpretation of hypoxia-and hypotension-related effects. Nevertheless, the observed breathing and heart rate changes strongly suggest that alterations in oxygen levels and blood pressure play a significant role in the observed seizure susceptibility. Increasing the sample size within each group is mandatory to improve statistical power and enhance result reliability. Future experiments should consider performing intensity sweeps by varying stimulation frequency, intensity, and duration at the beginning of the protocol to determine individual physiological thresholds. This approach would help set stimulation intensities appropriately, minimizing the impact on heart rate and breathing rate, while maintaining parameters that are associated with the anti-epileptic effect.

## Data Availability

The raw data supporting the conclusions of this article will be made available by the authors, without undue reservation.
