# Supplementary material for: The effect of vagus nerve stimulation on heart rate and respiration rate and their impact on seizure susceptibility in anaesthetized rats under pentylenetetrazol
Source: Front Neurosci. 2025 Mar 21;19:1487082. doi: 10.3389/fnins.2025.1487082 (PMC11968678; doi:10.3389/fnins.2025.1487082)
Supplement: Supplementary file 1 [file Data_Sheet_1.pdf]

Supplementary Material

1 Supplementary Figures and Tables

1.1 Supplementary Figures

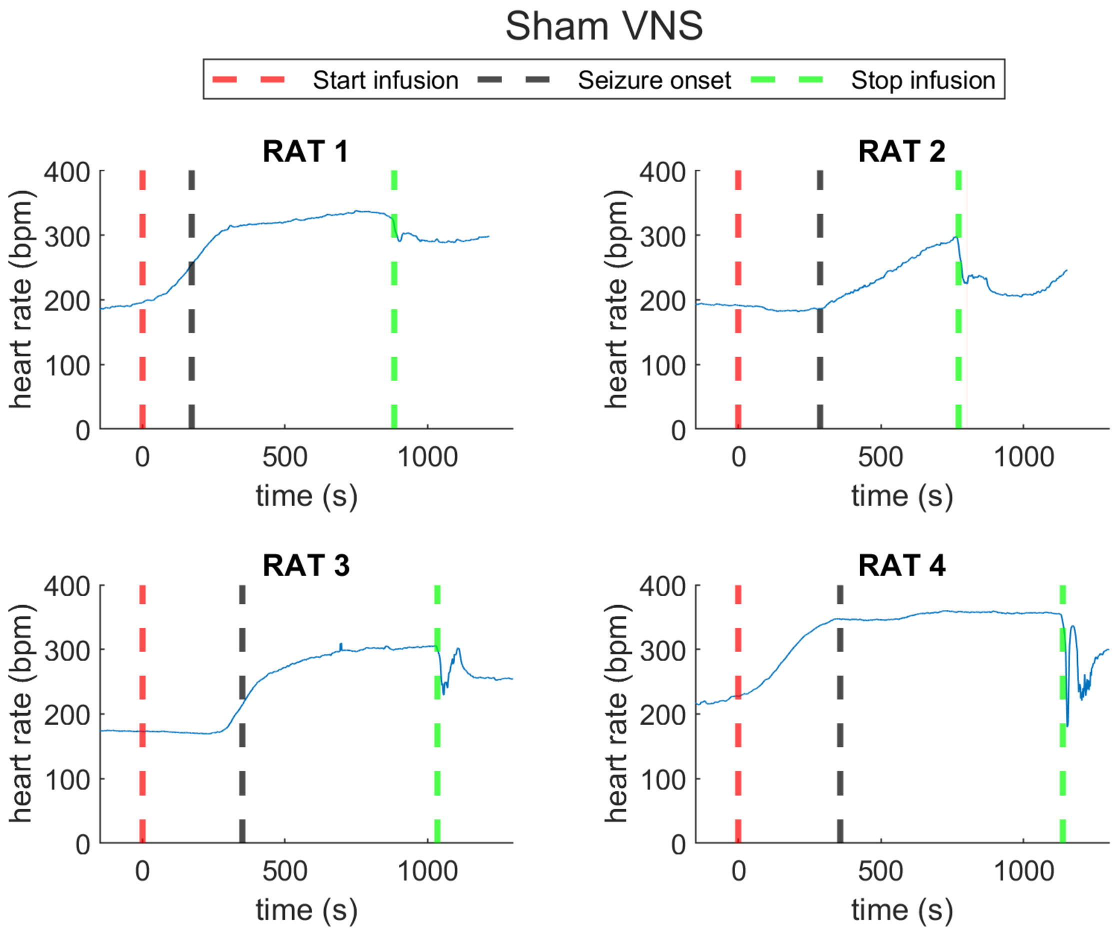

Supplementary Figure 1. Heart rate evolution for each rat in the sham stimulation group.

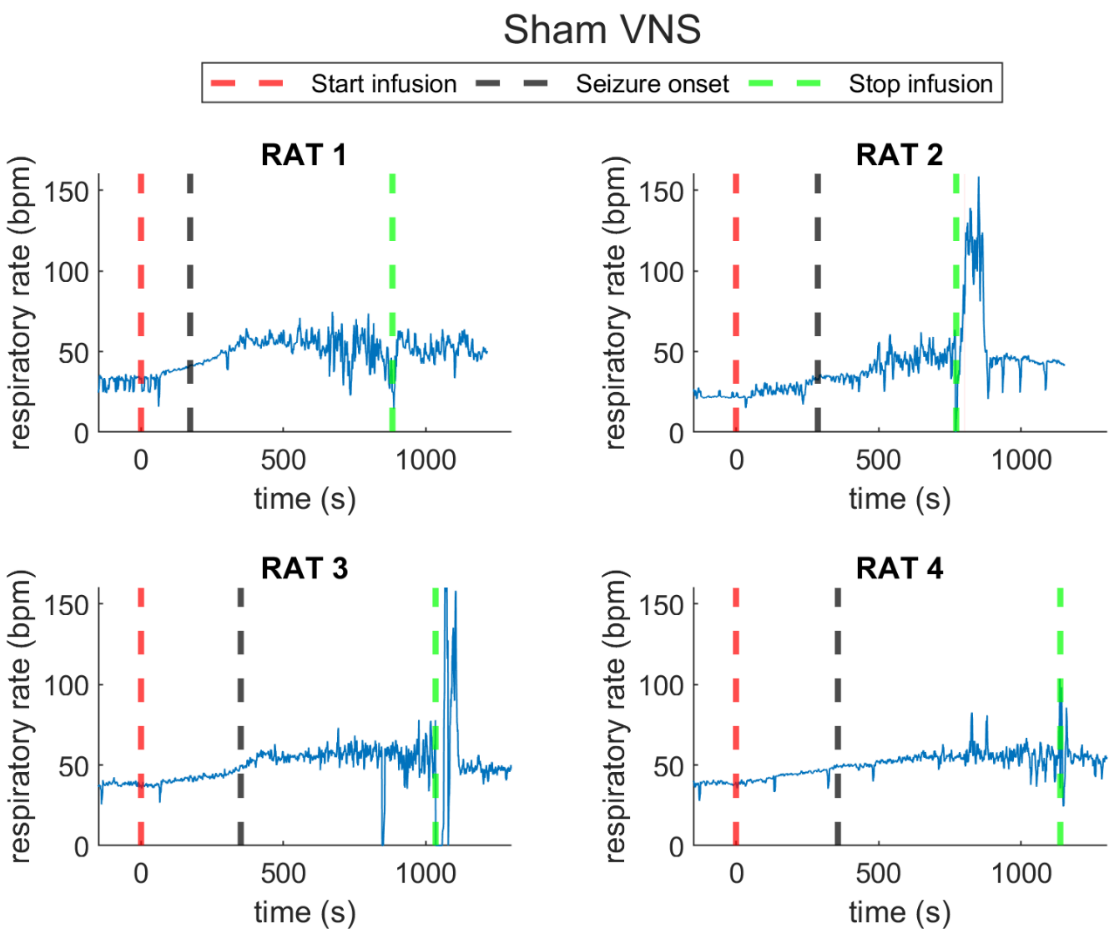

Supplementary Figure 2. Breathing rate evolution for each rat in the sham stimulation group.

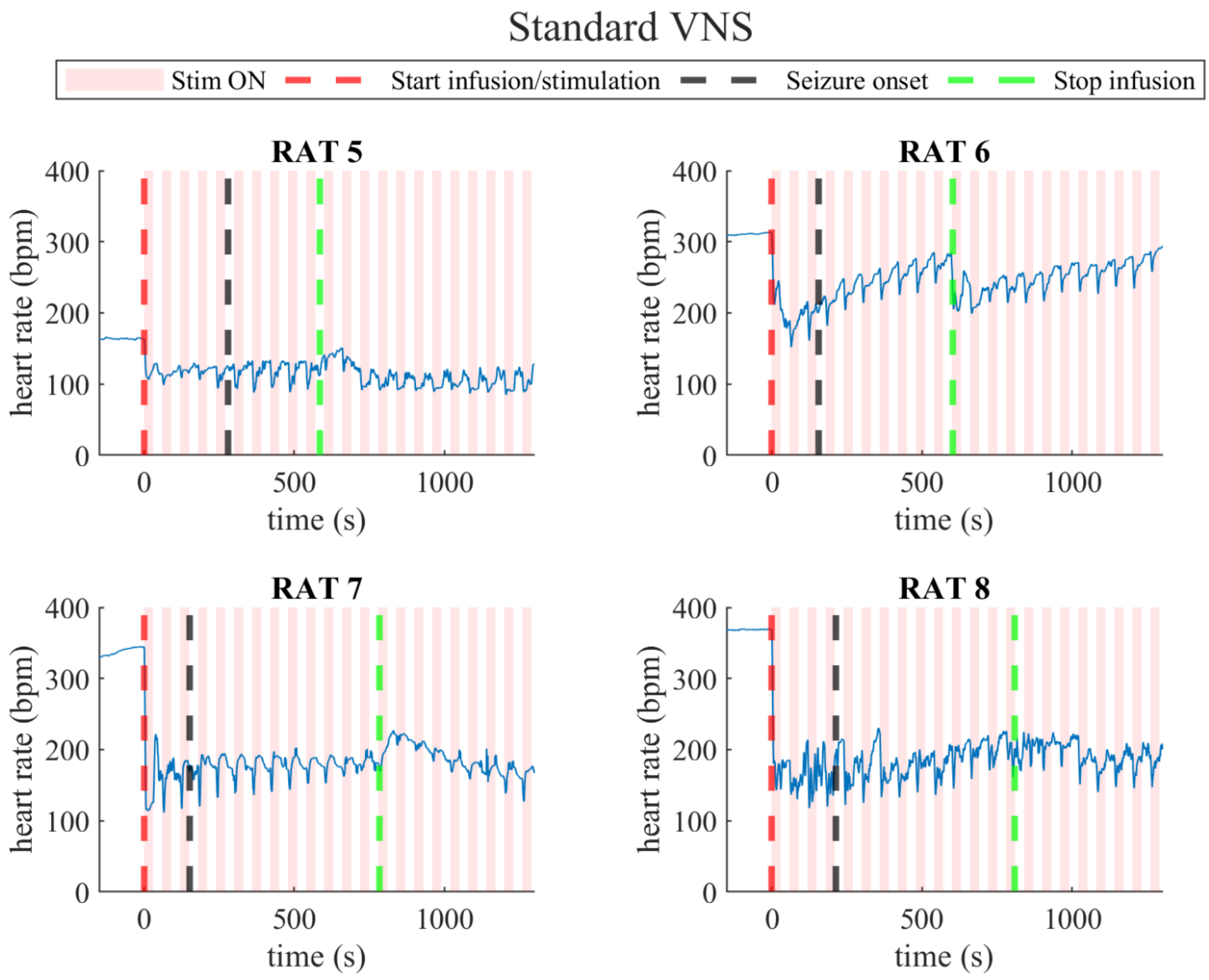

Supplementary Figure 3. Heart rate evolution for rats under standard VNS.

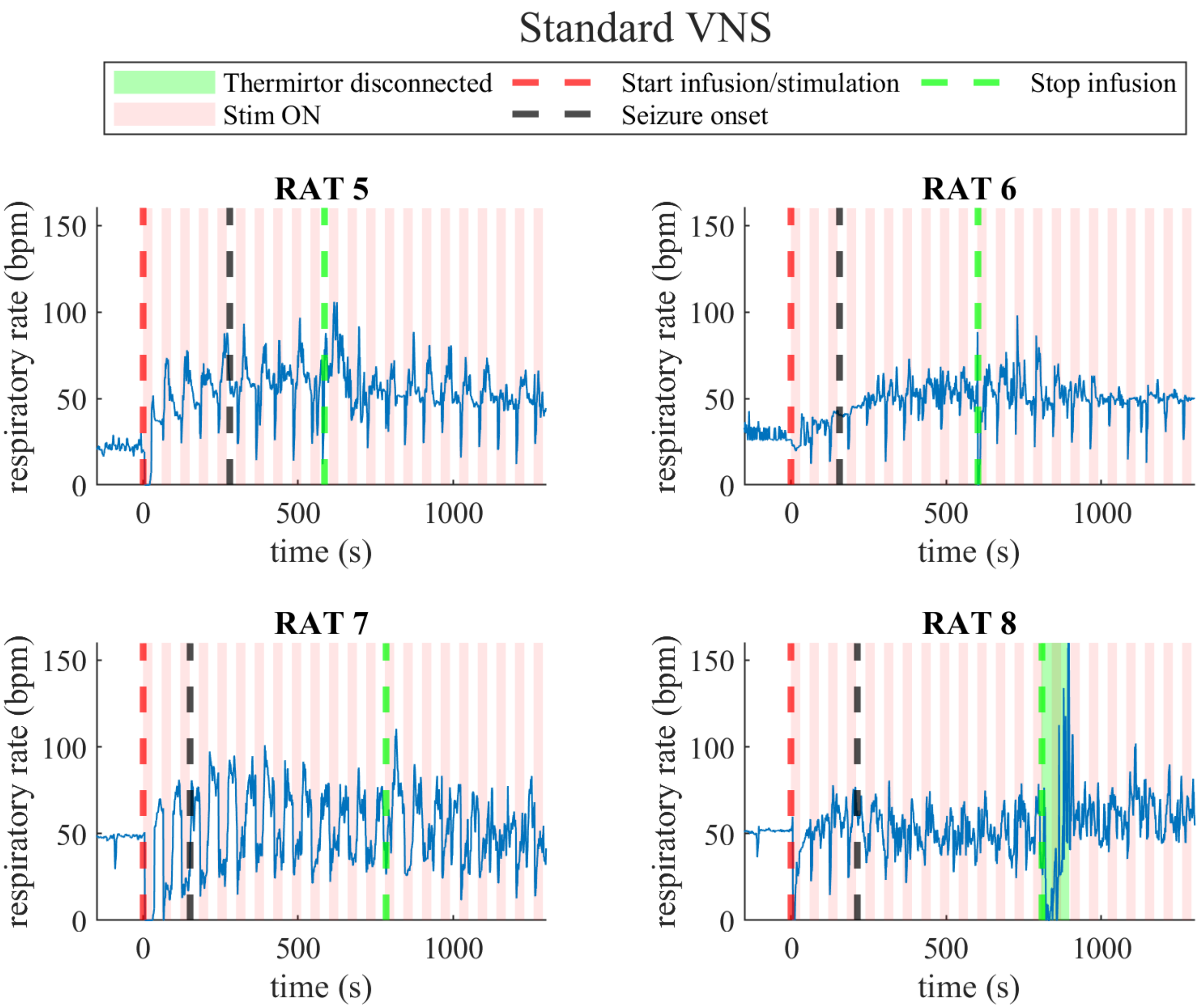

Supplementary Figure 4. Breathing rate evolution for rats under standard VNS.

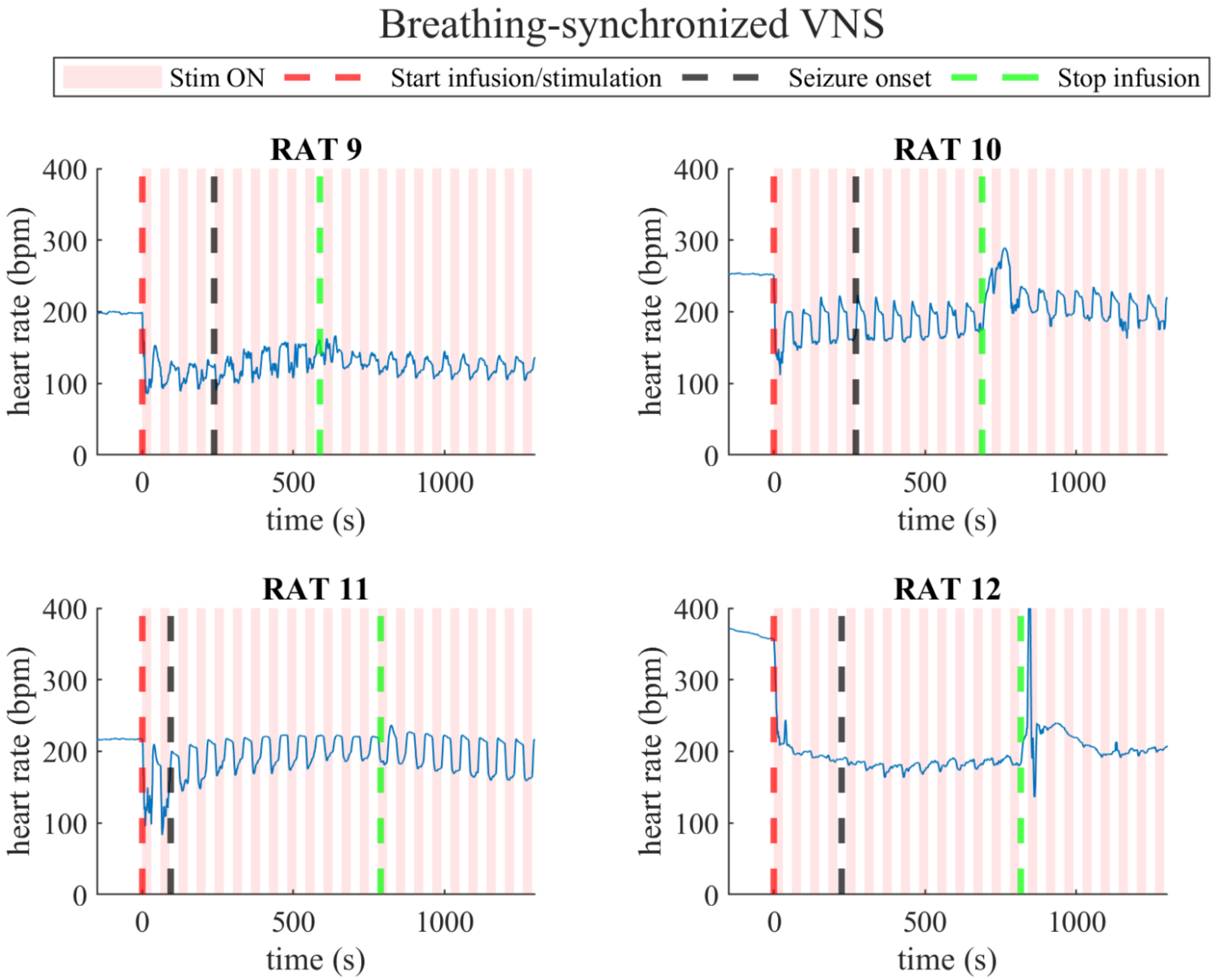

Supplementary Figure 5. Heart rate evolution in rats under breathing-synchronized VNS.

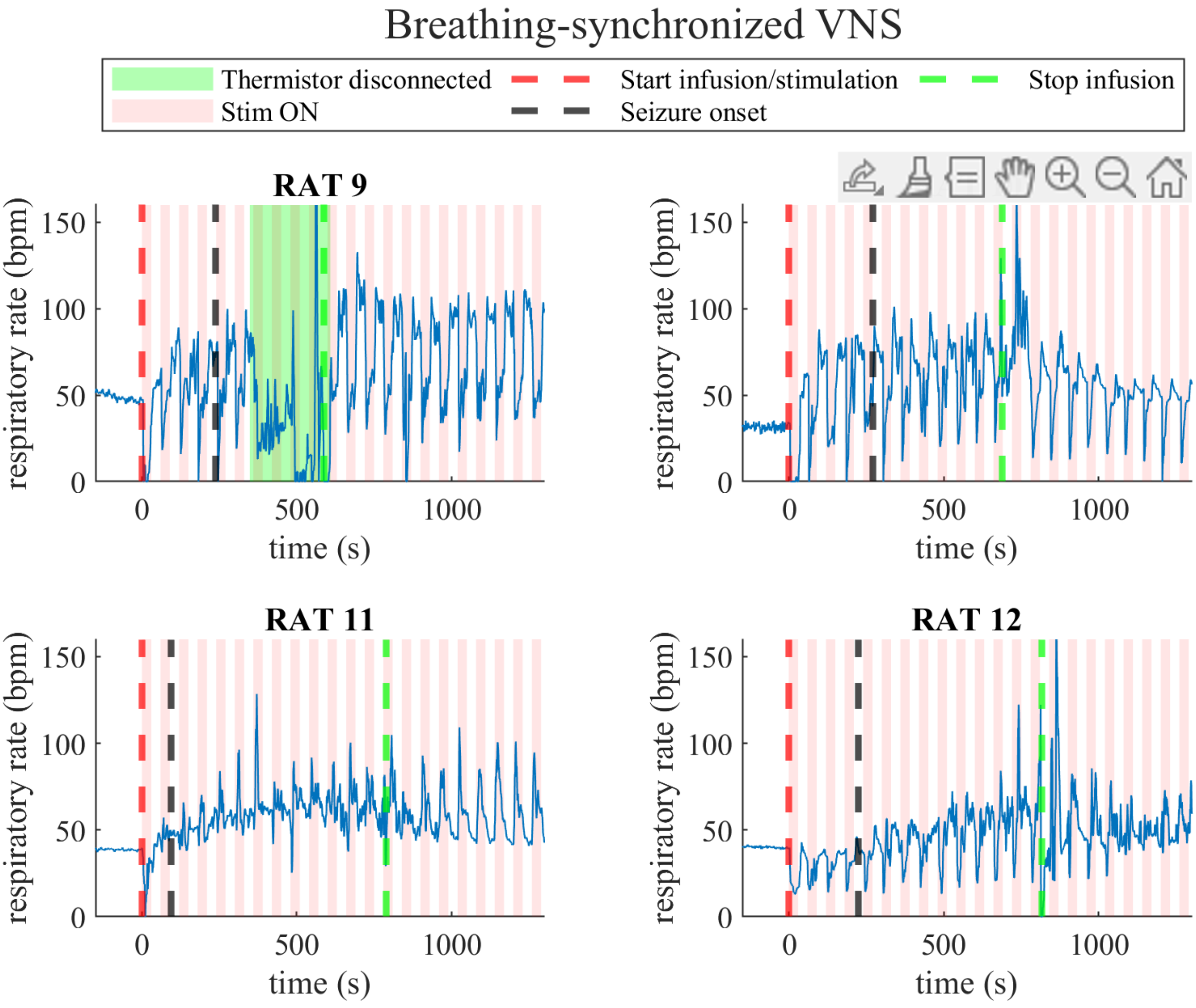

Supplementary Figure 6. Breathing rate evolution in rats under breathing-synchronized VNS.

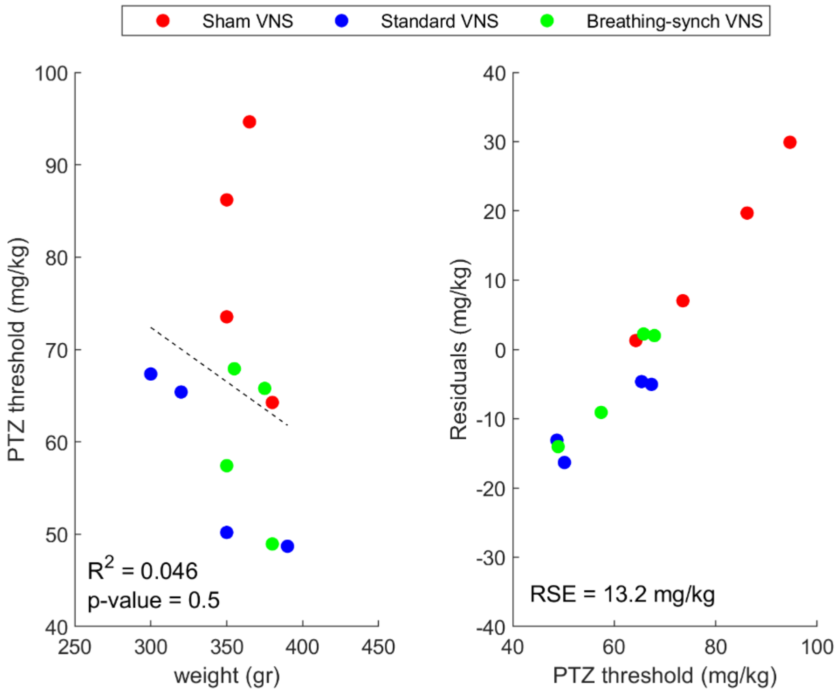

Supplementary Figure 7. Left: Linear regression between PTZ threshold and weight. Right: Residuals of the regression (RSE = Residual Standard Error).

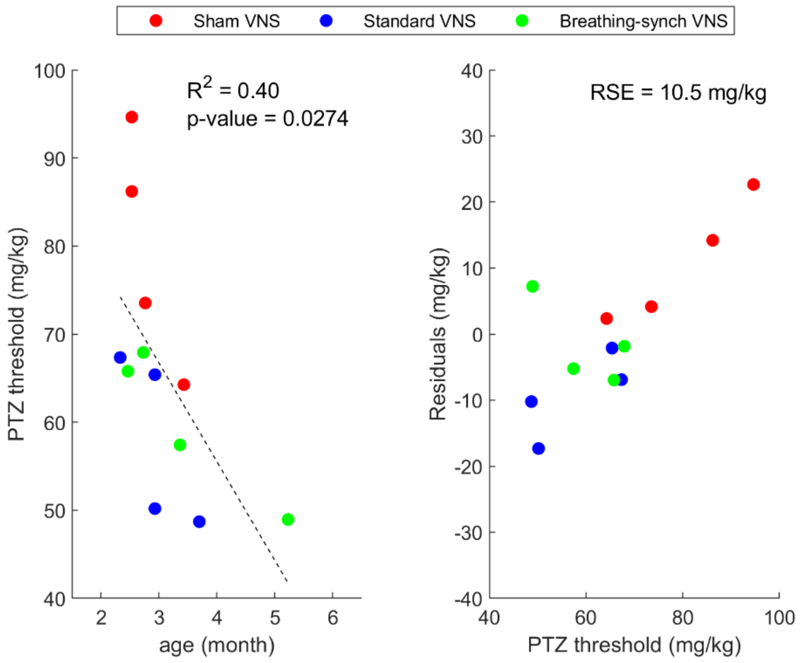

Supplementary Figure 8. Left: Linear regression between PTZ threshold and age. Right: Residuals of the regression (RSE = Residual Standard Error).

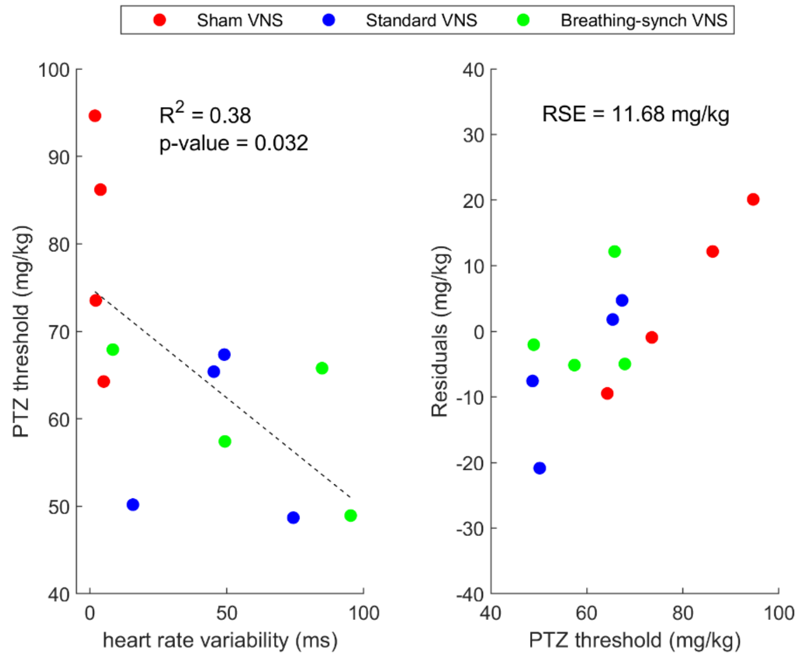

Supplementary Figure 9. Left: Linear regression between PTZ threshold and heart rate variability presented during the infusion. Right: Residuals of the regression (RSE = Residual Standard Error).

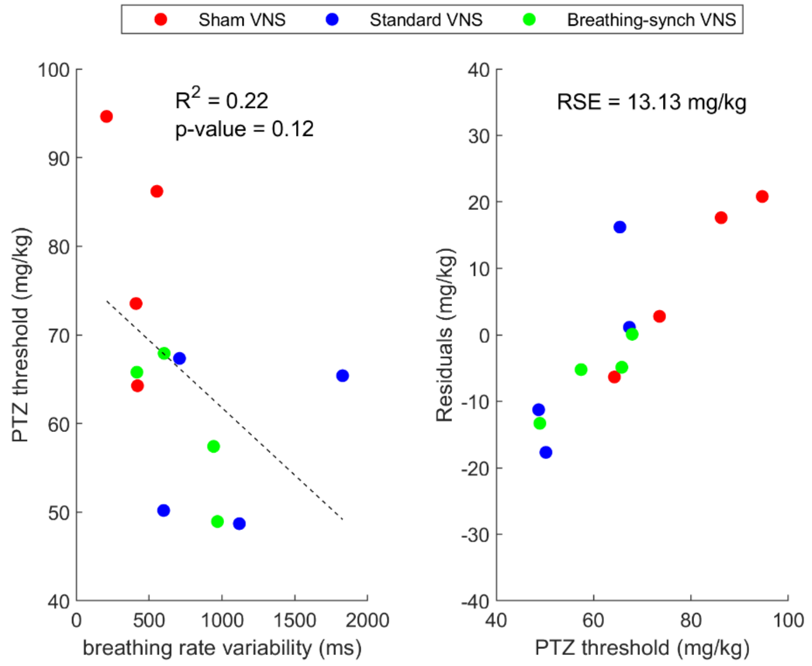

Supplementary Figure 10. Left: Linear regression between PTZ threshold and breathing rate variability presented during the infusion. Right: Residuals of the regression (RSE = Residual Standard Error).

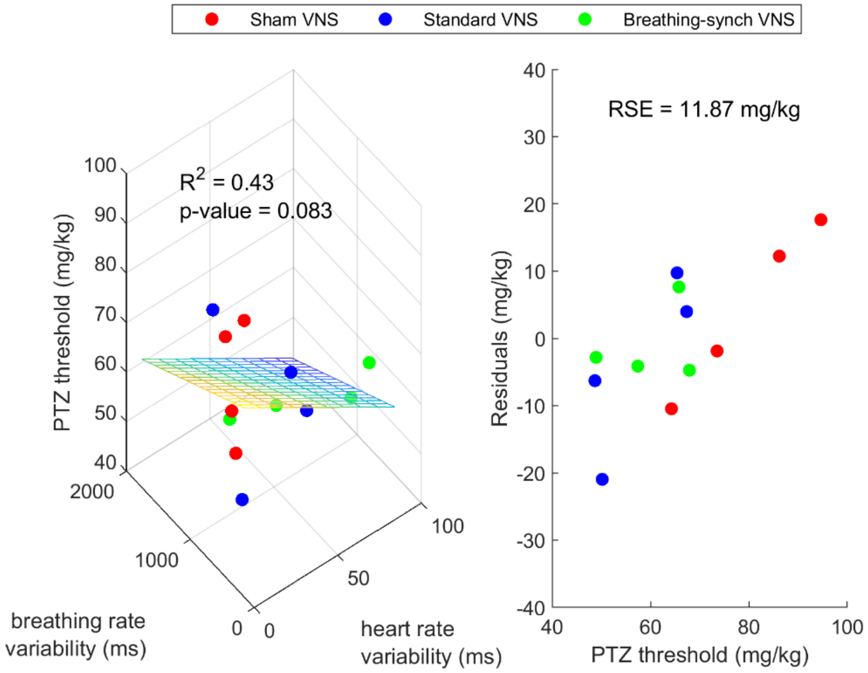

Supplementary Figure 11. Left: Linear regression between PTZ threshold and heart rate variability and breathing rate variability. Right: Residuals of the regression (RSE = Residual Standard Error).

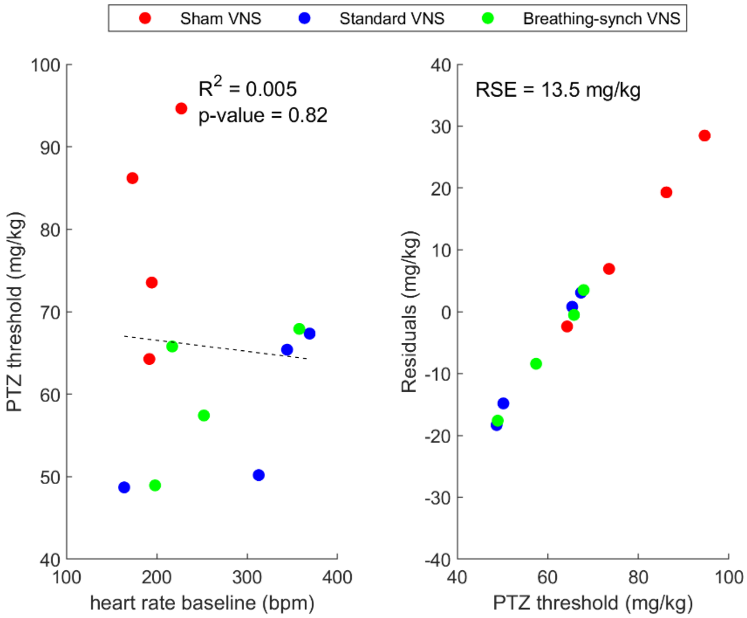

Supplementary Figure 12. Left: Linear regression between PTZ threshold and heart rate baseline values. Right: Residuals of the regression (RSE = Residual Standard Error).

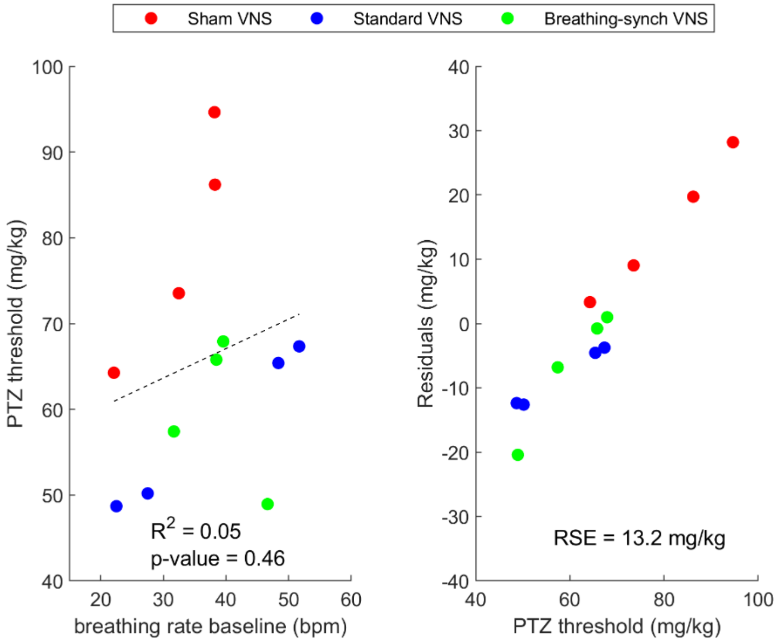

Supplementary Figure 13. Left: Linear regression between PTZ threshold and breathing rate baseline values. Right: Residuals of the regression (RSE = Residual Standard Error).

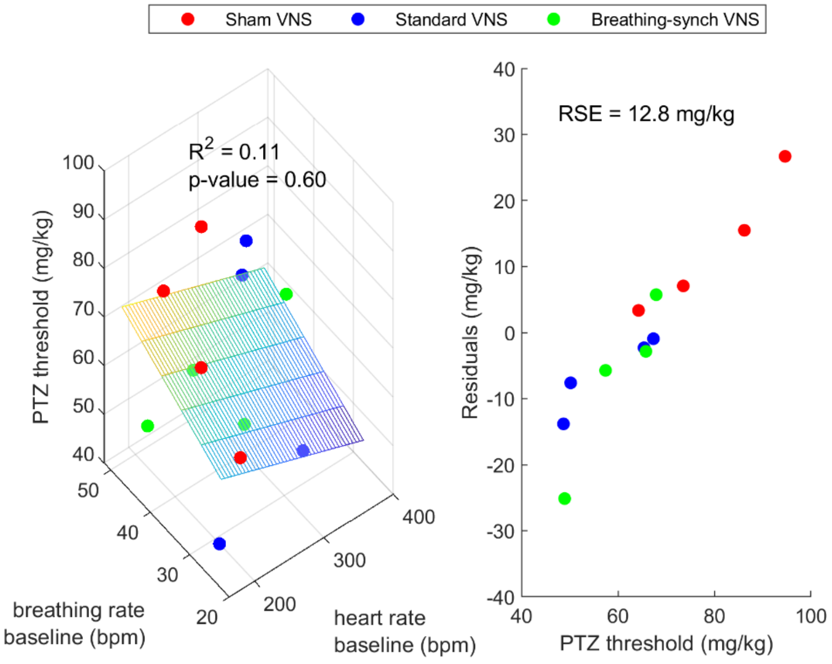

Supplementary Figure 14. Left: Linear regression between PTZ threshold and heart rate baseline and breathing rate baseline values. Right: Residuals of the regression (RSE = Residual Standard Error).

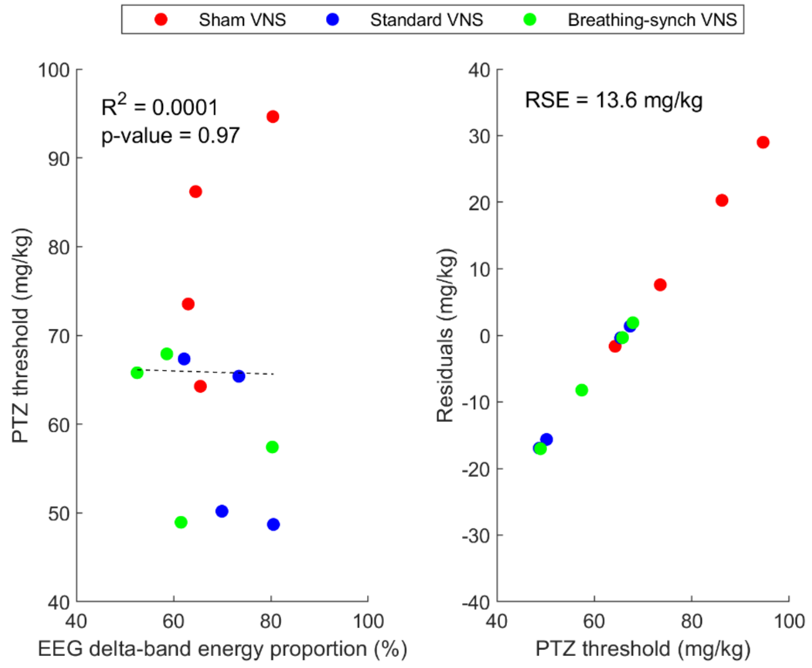

Supplementary Figure 15. Left: Linear regression between PTZ threshold and EEG delta-band energy proportion. Right: Residuals of the regression (RSE = Residual Standard Error).

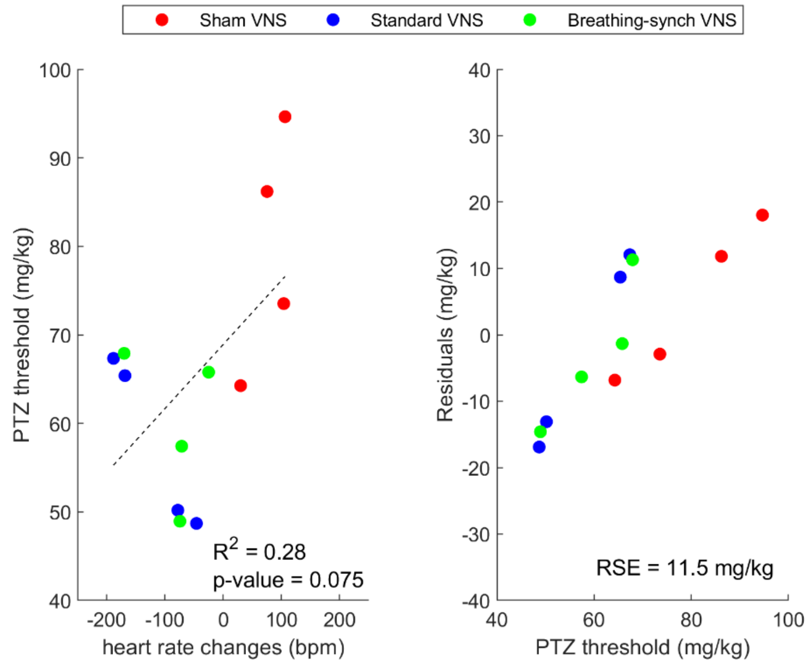

Supplementary Figure 16. Left: Linear regression between PTZ threshold and heart rate changes. Right: Residuals of the regression (RSE = Residual Standard Error).

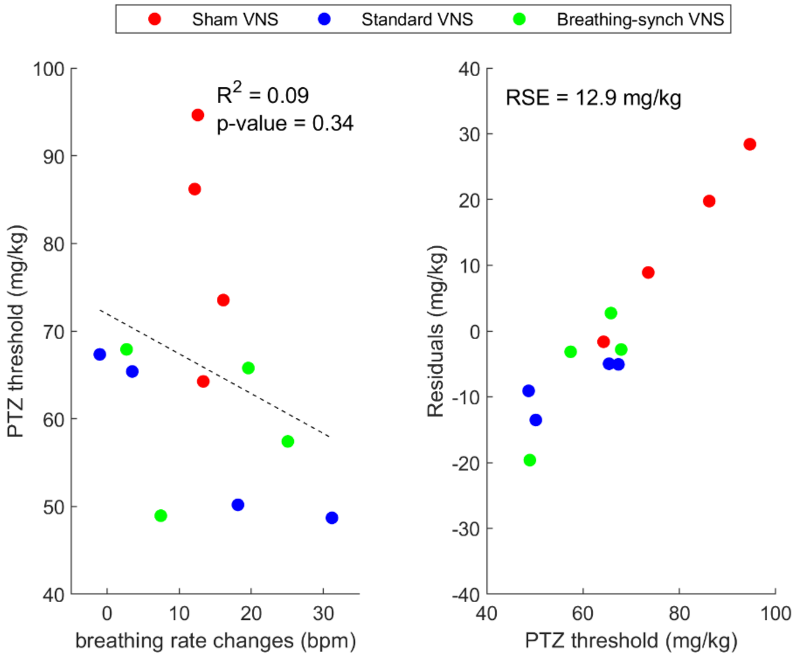

Supplementary Figure 17. Left: Linear regression between PTZ threshold and breathing rate changes. Right: Residuals of the regression (RSE = Residual Standard Error).

1.2 Supplementary Tables

**Sham VNS**	Weight (gr)	Age (month)	**Standard VNS**	Weight (gr)	Age (month)	**Breathing-synchronized VNS**	Weight (gr)	Age (month)
Rat 1	350	2.8	Rat 5	390	3.7	Rat 9	380	5.2
Rat 2	380	3.4	Rat 6	350	2.9	Rat 10	350	3.4
Rat 3	350	2.5	Rat 7	320	2.9	Rat 11	375	2.5
Rat 4	365	2.5	Rat 8	300	2.3	Rat 12	355	2.7
**Average ± SD**	361.3 ± 14.4	2.8 ± 0.4	**Average ± SD**	340.0 ± 39.2	3.0 ± 0.6	**Average ± SD**	365.0 ± 14.7	3.5 ± 1.2

Supplementary Table 1. Weight and age of the rats. The total average weight was 355.4 ± 25.8 grams, and for age was 3.1 ± 0.8 months.

	Sham VNS				Standard VNS				Breathing-synchronized VNS				
	Rat 1	Rat 2	Rat 3	Rat 4	Rat 5	Rat 6	Rat 7	Rat 8	Rat 9	Rat 10	Rat 11	Rat 12	**Average ± SD**
Phase Locking Index	0.79	0.77	0.81	0.90	0.8	0.73	0.93	0.94	0.93	0.95	0.81	0.89	0.86 ± 0.08

Supplementary Table 2. Phase Locking Index between the VENG envelope and the respiration signal for each rat.

	Sham VNS				Standard VNS				Breathing-synchronized VNS				
	Rat 1	Rat 2	Rat 3	Rat 4	Rat 5	Rat 6	Rat 7	Rat 8	Rat 9	Rat 10	Rat 11	Rat 12	**Average ± SD**
Preictal	64.3	71.4	74.0	81.9	81.9	77.9	78.3	75.8	67.9	74.7	60.5	70.2	73.2 ± 6.7
Baseline	63.0	65.5	64.5	80.4	80.5	69.9	73.4	62.2	61.5	80.3	52.5	58.6	67.7 ± 9.3
Difference	1.3	5.9	9.5	1.5	1.3	8.0	4.9	13.7	6.4	-5.6	8.0	11.6	5.5 ± 5.3

Supplementary Table 3. EEG delta-band energy proportion (%) per rat in baseline and preictal periods.

	Heart rate (bpm)			
	Baseline	Infusion	Difference	Post-infusion
Rat 1	194.5	298.8	+104.3	291.8
Rat 2	191.8	222.0	+30.2	208.7
Rat 3	173.1	248.8	+75.7	254.0
Rat 4	227.2	333.8	+106.6	310.9
**Average ± SD**	**196.6 ± 22.5**	**275.8 ± 50.0**	**+79.2 ± 35.5**	**266.3 ± 45.1**

Supplementary Table 4. Mean heart rate per rat in the sham stimulation group. The difference is always with respect to the baseline values.

	Breathing rate (bpm)			
	Baseline	Infusion	Difference	Post-infusion
Rat 1	32.5	48.6	+16.1	52.0
Rat 2	22.1	35.5	+13.3	44.0
Rat 3	38.2	50.4	+12.2	47.1
Rat 4	38.2	50.8	+12.6	52.5
**Average ± SD**	**32.8 ± 7.6**	**46.3 ± 7.3**	**+13.6 ± 1.8**	**48.9 ± 4.1**

Supplementary Table 5. Mean breathing rate per rat in the sham stimulation group. The difference is always with respect to the baseline values.

	Heart rate (bpm)							
	Baseline	Infusion	Difference	ON period	Difference	OFF period	Difference	Post-infusion
Rat 5	164.0	118.4	-45.6	113.0	-51.0	124.4	-39.5	105.3
Rat 6	312.8	235.2	-77.7	228.70	-84.1	241.7	-71.1	242.0
Rat 7	344.2	175.6	-168.5	173.8	-170.4	177.5	-166.7	185.6
Rat 8	369.1	180.9	-188.2	171.1	-198.0	191.8	-177.3	189.2
**Average ± SD**	**297.5 ± 92.0**	**177.5 ± 47.7**	**-120.0 ± 69.1**	**171.6 ± 47.3**	**-125.9 ± 69.6**	**183.9 ± 48.3**	**-113.7 ± 68.7**	**180.5 ± 56.4**

Supplementary Table 6. Mean heart rate per rat in the standard VNS stimulation group. The difference is always with respect to the baseline values.

	Breathing rate (bpm)							
	Baseline	Infusion	Difference	ON period	Difference	OFF period	Difference	Post-infusion
Rat 5	22.5	53.7	+31.2	51.3	+28.8	56.2	+33.7	53.5
Rat 6	27.5	45.7	+18.2	44.5	+16.9	46.9	+19.4	53.6
Rat 7	48.4	51.9	+3.5	34.8	-13.6	68.8	+20.4	51.8
Rat 8	51.7	50.7	-1.0	50.4	-1.3	51.2	-0.5	59.2
**Average ± SD**	**37.5±14.6**	**50.5±3.4**	**+13.0±14.6**	**45.2±7.6**	**+7.7±18.8**	**55.8±9.5**	**+18.2±14.1**	**54.5 ± 3.2**

Supplementary Table 7. Mean breathing rate per rat in the standard VNS stimulation group. The difference is always with respect to the baseline values.

	Heart rate (bpm)							
	Baseline	Infusion	Difference	ON period	Difference	OFF period	Difference	Post-infusion
Rat 9	198.1	124.1	-74.0	115.1	-83.0	135.5	-62.6	126.6
Rat 10	252.1	181.1	-71.0	165.6	-86.5	196.5	-55.6	209.4
Rat 11	217.1	192.4	-24.7	175.9	-41.2	209.1	-8.0	195.8
Rat 12	357.7	187.8	-169.9	186.5	-171.2	189.1	-168.6	204.5
**Average ± SD**	**256.2±71.2**	**171.3±31.9**	**-84.9±61.0**	**160.8±31.6**	**-95.5±54.5**	**182.5±32.4**	**-73.7±67.8**	**184.1 ± 38.7**

Supplementary Table 8. Mean heart rate per rat in the breathing-synchronized VNS stimulation group. The difference is always with respect to the baseline values.

	Breathing rate (bpm)							
	Baseline	Infusion	Difference	ON period	Difference	OFF period	Difference	Post-infusion
Rat 9	46.6	54.1	+7.5	43.6	-3.0	69.0	+22.4	36.8
Rat 10	31.7	56.8	+25.1	42.9	+11.2	71.2	+39.5	56.3
Rat 11	38.5	58.1	+19.6	59.5	+21.0	56.7	+18.2	58.0
Rat 12	39.6	42.3	+2.7	36.8	-2.8	48.4	+8.8	49.3
**Average ± SD**	**39.1±6.1**	**52.8±7.2**	**+13.7±10.4**	**45.7±9.7**	**+6.6±11.7**	**61.3±10.7**	**+22.2±12.8**	**50.1 ± 9.7**

Supplementary Table 9. Mean breathing rate per rat in the breathing-synchronized VNS stimulation group. The difference is always with respect to the baseline values.

	Stimulation onset														
	1	2	3	4	5	6	7	8	9	10	11	12	13	14	**Average ± SD**
Rat 5	22.7	3.2	3.3	3.5	3.5	3.9	4.2	3.7	4.3	4.0	/	/	/	/	5.6 ± 6.0
Rat 6	3.1	4.9	4.4	4.3	-	4.5	3.4	-	3.5	-	/	/	/	/	4.0 ± 0.7
Rat 7	-	7.2	4.6	4.4	3.6	4.0	3.1	3.4	-	3.8	3.7	3.1	/	/	4.1 ± 1.2
Rat 8	6.3	4.1	4.1	3.9	-	-	-	-	-	-	-	-	3.4	*	4.4 ± 1.1
Rat 9	13.0	3.3	3.8	5.9	6.8	3.2	*	*	*	*	/	/	/	/	6.0 ± 3.8
Rat 10	22.7	7.4	3.1	7.3	-	5.9	5.1	-	3.2	-	-	/	/	/	7.8 ± 6.8
Rat 11	7.4	-	-	-	-	-	-	-	3.6	-	-	-	/	/	5.5 ± 2.6
Rat 12	4.6	3.9	4.2	4.1	4.2	4.5	3.5	3.6	4.5	4.2	3.1	-	-	3.3	4.0 ± 0.5

Supplementary Table 10. Maximal apnea duration (seconds) per rat per stimulation onsets. The underlined numbers represent the outlier detected in apnea duration. The symbol (/) means that no apnea detection was performed because the infusion was stopped. The symbol (*) means that no apnea was detected due to the detachment of the thermistor. The symbol (-) means that no apnea was detected during the stimulation onset.

**Sham VNS**	Heart rate variability (ms)	Breathing rate variability (ms)	**Standard VNS**	Heart rate variability (ms)	Breathing rate variability (ms)	**Breathing-synch VNS**	Heart rate variability (ms)	Breathing rate variability (ms)
Rat 1	2.2	410.3	Rat 5	74.3	1119.4	Rat 9	95.2	969.3
Rat 2	5.1	420.5	Rat 6	15.7	599.9	Rat 10	49.3	942.8
Rat 3	3.9	553.3	Rat 7	45.2	1827.9	Rat 11	84.8	416.2
Rat 4	1.9	207.9	Rat 8	49.1	708.9	Rat 12	8.4	603.2
**Average ± SD**	3.2 ± 1.5	398.0 ± 142.5	**Average ± SD**	46.1 ± 24.0	1064.0 ± 556.2	**Average ± SD**	59.4 ± 39.3	732.9 ± 269.0

Supplementary Table 11. Heart rate and breathing rate variability (RMSSD) per rat during infusion.

**Sham VNS**	PTZ threshold (mg/kg)	**Standard VNS**	PTZ threshold (mg/kg)	**Breathing-synch VNS**	PTZ threshold (mg/kg)
Rat 1	73.5	Rat 5	48.7	Rat 9	48.9
Rat 2	64.3	Rat 6	50.2	Rat 10	57.4
Rat 3	86.2	Rat 7	65.4	Rat 11	65.8
Rat 4	94.6	Rat 8	67.3	Rat 12	67.9
**Average ± SD**	79.7 ± 13.4	**Average ± SD**	57.9 ± 9.8	**Average ± SD**	60.0 ± 8.7

Supplementary Table 12. PTZ threshold per rat.

		Latency (s)	Ictal duration (s)	Post-ictal duration (s)
Sham VNS	Rat 1	173.9	708.5	34.3
	Rat 2	286.5	484.6	103.8
	Rat 3	349.1	685.3	80.1
	Rat 4	356.4	779.3	37.4
	**Average ± SD**	**291.5 ± 84.4**	**664.4 ± 126.4**	**63.9 ± 33.8**
Standard VNS	Rat 5	279.4	304.9	112.7
	Rat 6	156.6	445.7	45.1
	Rat 7	152.8	631.9	54.6
	Rat 8	213.3	594.8	54.6
	**Average ± SD**	**200.5 ± 59.5**	**494.3 ± 149.7**	**66.7 ± 31.0**
Breathing-synchronized VNS	Rat 9	118.3	469.0	43.2
	Rat 10	215.6	473.4	90.7
	Rat 11	215.5	573.9	37.2
	Rat 12	278.1	536.8	64.0
	**Average ± SD**	**206.9 ± 66.0**	**513.3 ± 50.9**	**58.8 ± 24.2**

Supplementary Table 13. Latency, Ictal duration, and post-ictal duration for each rat.
